# MicroRNAs in Tumor Cell Metabolism: Roles and Therapeutic Opportunities

**DOI:** 10.3389/fonc.2019.01404

**Published:** 2019-12-11

**Authors:** Abraham Pedroza-Torres, Sandra L. Romero-Córdoba, Montserrat Justo-Garrido, Iván Salido-Guadarrama, Rubén Rodríguez-Bautista, Sarita Montaño, Rodolfo Muñiz-Mendoza, Cristian Arriaga-Canon, Verónica Fragoso-Ontiveros, Rosa María Álvarez-Gómez, Greco Hernández, Luis A. Herrera

**Affiliations:** ^1^Cátedra CONACyT-Clínica de Cáncer Hereditario, Instituto Nacional de Cancerología, Mexico City, Mexico; ^2^Departamento de Bioquímica, Instituto Nacional de Ciencias Médicas y Nutrición “Salvador Zubirán”, Mexico City, Mexico; ^3^Unidad de Investigación Biomédica en Cáncer, Instituto Nacional de Cancerología - Instituto de Investigaciones Biomédicas - Universidad Nacional Autónoma de México (UNAM), Mexico City, Mexico; ^4^Biología Computacional, Instituto Nacional de Enfermedades Respiratorias “Ismael Cosío Villegas”, Mexico City, Mexico; ^5^Unidad de Oncología Torácica y Laboratorio de Medicina Personalizada, Instituto Nacional de Cancerologia, Mexico City, Mexico; ^6^Laboratorio de Bioinformática, Facultad de Ciencias Químico-Biológicas, Universidad Autónoma de Sinaloa (FCQB-UAS), Culiacán, Mexico; ^7^Clínica de Cáncer Hereditario, Instituto Nacional de Cancerología, Mexico City, Mexico; ^8^Laboratorio de Traducción y Cáncer, Unidad de Investigaciones Biomedicas en Cáncer, Instituto Nacional de Cancerolgía, Mexico City, Mexico

**Keywords:** microRNAs, reprogramming metabolism, regulation, therapeutic targets, tumor cell metabolism

## Abstract

Dysregulated metabolism is a common feature of cancer cells and is considered a hallmark of cancer. Altered tumor-metabolism confers an adaptive advantage to cancer cells to fulfill the high energetic requirements for the maintenance of high proliferation rates, similarly, reprogramming metabolism confers the ability to grow at low oxygen concentrations and to use alternative carbon sources. These phenomena result from the dysregulated expression of diverse genes, including those encoding microRNAs (miRNAs) which are involved in several metabolic and tumorigenic pathways through its post-transcriptional-regulatory activity. Further, the identification of key actionable altered miRNA has allowed to propose novel targeted therapies to modulated tumor-metabolism. In this review, we discussed the different roles of miRNAs in cancer cell metabolism and novel miRNA-based strategies designed to target the metabolic machinery in human cancer.

## Introduction

Ever since their discovery in 1993 (1), microRNAs emerged as a new class of small RNAs with a critical role in the regulation of gene expression. MicroRNAs (miRNAs or miRs) are endogenous small non-coding RNAs from 18-25 nucleotides in length that regulate gene expression via base complementarity between the seed region of the microRNA and the 3′-untranslated region (UTR) of the target mRNA (1, 2). Depending on the degree of complementarity, miRNAs binding can induce mRNA degradation, translational repression, or both (3–5). For the considerable relevance of miRNAs in gene expression, these tiny RNA molecules have recently been called “master regulators of gene expression” (6).

The biogenesis of miRNAs has been extensively studied (7–9). For instance, genes encoding miRNAs show distinct genomic locations, such as intergenic, intronic, exonic, or mirtronic (a type of miRNA that is located in the introns of the mRNAs). Genes encoding miRNAs are transcribed in the nucleus in the form of long primary transcripts (pri-miRNAs) typically, although not exclusively, by RNA Pol II (10). Afterward, pri-miRNAs are processed into a small stem-loop transcript of approximately 55-70 nucleotides by the RNA-binding protein DiGeorge Syndrome Critical Region 8 (DGCR8) and Drosha (a ribonuclease III enzyme) (11, 12). This new structure, termed pre-miRNA, is recognized by Exportin 5 (Exp 5) and is exported from the nucleus to the cytoplasm (13). Once in the cytoplasm, pre-miRNA hairpins are cleaved by the Dicer RNase III enzyme and TRBP (TAR RNA-binding protein), resulting in a ~22 nucleotide mature miRNA-miRNA^*^ duplex (14–16).

Finally, the mature miRNA is loaded onto Argonaute 2 protein (AGO2) and the RNA-induced silencing complex (RISC) to catalyze site-specific cleavage or translational repression of their mRNA targets (17, 18). The post-transcriptional regulation of gene expression by miRNAs is of paramount importance, thus, it is estimated that miRNAs could regulate nearly 60% of all human protein-coding genes (19). miRNAs are involved in several cellular processes, such as proliferation, development, differentiation, apoptosis, carcinogenesis, and energy metabolism (20–26).

During tumorigenesis, dysregulated metabolism represents an adaptive advantage of cancer cell that promote uncontrolled cell division, cell growth, and survival (27, 28). One of the best characterized metabolic disorders during cancer development is the Warburg effect, that increase glucose uptake and lactate production. During the Warburg effect, miRNAs activity contributes to keeping high levels of glycolysis. miRNAs also control other crucial steps of the energy metabolism, including glucose transport, glycolysis, tricarboxylic acid cycle, glutaminolysis, altered lipid metabolism, insulin production, cholesterol, and lipid homeostasis, as well as amino acid and nucleotide biogenesis (29–33).

In this review, we focus on the different roles of miRNAs in cancer metabolism and discuss novel miRNA-based strategies designed to target different processes in human cancer. We also explore the links between microbiota and miRNA networks and cancer, with a particular focus on genotoxicity and tumor-metabolism.

## Metabolic Reprogramming in Cancer Cells

Upon cancer onset and progression, cells exhibit various growth, proliferation, and survival phenotypes. These cancer hallmarks are supported by a catabolic and anabolic metabolism reprogramming. Increasing evidence has shown that metabolic changes are the result of complex processes, and several cellular pathways are implicated (34–36). Recent findings have led to a significant shift in our understanding of altered metabolic states, which now are seen as a central transformative force in cancer development (37–39).

The Warburg effect is thought to be an early event in cancer that promotes rapid adaptation to higher bioenergetic demands, such as, excessive proliferation and hypoxic microenvironments. Warburg effect is characterized by: (a) supports the demand for ATP synthesis and promotes its flux into biosynthetic pathways to achieve an uncontrolled proliferation; (b) maintains an acidic microenvironment via the accumulation of lactate; and (c) allows for ROS signaling homeostasis (40–43). Moreover, reprogramming energy metabolism promotes tumor cells to use alternative carbon sources such as glutamine, considered to be the second source of nutrients after glucose. Glutamine is the most abundant amino acid in cells, and its catabolism results in several amounts of cellular precursors, including glutamate, aspartate, pyruvate, lactate, alanine, and citrate (44–46).

For many years, the Warburg effect was considered as a synonym for metabolic reprogramming. However, it is clear that this phenotype alone cannot explain all the metabolic alterations that enhance the formation of primary tumors and their development throughout invasion and metastasis. Recent publications have also reported the metabolic interactions between tumors and the microenvironment involving cancer-associated fibroblasts, immune cells, and microbiota, which allows us to expand our understanding of the metabolic reprogramming and reveals the complex interaction networks required to establish the tumor phenotype (47–49). Most of the aforementioned metabolic features are a consequence of the deregulation of several cell pathways and often involve altered oncogenes, tumor suppressors, and miRNA.

## miRNAs Regulation of Metabolic Pathways in Cancer

In the last decade, a growing volume of evidence has revealed the role of miRNAs in the regulation of energy metabolism, directly, through the regulation of glucose transporters (GLUT family), enzymes (hexokinase 1/2, Aldolase A), and protein kinases (AMPK, PI3K), or indirectly, through inhibition of several transcriptional factors (p53, c-Myc) (50–52). In any case, the role of miRNAs in the regulation of energy metabolism has gained much interest by their nature to modulate cellular metabolism and the possibility to use miRNAs-targets genes circuits as cancer therapies. Therefore, we review the main pathways of energy metabolism, the genes involved in each metabolic signaling and their transcriptional landscapes articulated by the miRNAs in cancer programs.

## miRNAs and Glucose Transporters

Glucose represents the main source of cellular energy. In cancer, tumor cells increase their glucose consumption to maintain the high energy requirements. However, due to the hydrophilic composition of glucose, it is not able to cross the plasma membrane by its own. To overcome this situation, tumor cells induce the expression of several members of the glucose transporters family (GLUTs, also named SLC2A proteins). Glucose transporters are membrane-associated carrier proteins responsible for facilitating the transport of glucose across the plasma membrane. In the human genome, 14 GLUT proteins have been found. Among different members of the GLUTs family, the expression of GLUT1, GLUT2, and GLUT3 has been reported to be upregulated in different types of tumors, whereas GLUT4 and GLUT5 are downregulated (53, 54). miRNAs control glucose uptake by regulating the GLUTs expression; for example, miR-144 and miR-132 are two miRNAs that have been associated with the regulation of GLUT1, one of the most broadly expressed isoforms in various cell types. Lui et al. reported that the downregulation of miR-144 induces an increase in glucose uptake in lung cancer (55). Moreover, Qu et al. demonstrated that the decrease in miR-132 expression altered glucose metabolism in prostate cancer (56). Additionally, miR-150 has been reported as a GLUT1 regulator in CD4+ cells (57). In renal cell carcinoma, miR-138, miR-150, miR-199a-3p, and miR-532-5p overexpression are associated with a decreased expression of GLUT 1, whereas miR-19a, miR-19b, miR-130b, and miR-301a decrease are directly associated with an over-expression of GLUT 1 (58).

GLUT3, another member of the glucose transport proteins family, is also regulated by miRNAs. Fei et al. demonstrated that miR-195-5p directly regulates the expression of GLUT3, and consequently decreases glucose uptake and inhibits cell growth in T24 bladder cancer cells (59). Similar results were reported by Dai DW in U251 and LN229 glioblastoma cells through the activity of miR-106a over GLUT3. Additionally, the authors indicated that miR-106a down-regulation is associated with glioblastoma patients survival (60).

Other examples of miRNAs that regulate glucose uptake are miR-233 and miR-133, which directly regulate the expression of GLUT4 (26, 61). Interestingly, miR-21 and miR-23a indirectly regulate the expression of GLUT4, as a result of their regulation over two GLUT4 translocators: PTEN and SMAD4 (62, 63). An exhaustive work published by Esteves et al., highlight the role of miR-21a-5p, miR-29a-3p, miR-29c-3p, miR-93-5p, miR-106b-5p, miR-133a-3p, miR-133b-3p, miR-222-3p, and miR-223-3p that directly or indirectly regulate the expression of GLUT4 (64). To our knowledge, there are no reports describing other members of the GLUT family regulated by miRNAs, although miRNA target prediction analysis identifies a set of miRNAs capable to silence them; however, further studies are needed to determine their contribution to aberrant tumor cell metabolism.

## miRNAs in Glycolysis

Unlike tumor cells, normal cells obtain energy in the form of ATP through the glucose-derived pyruvate by the mitochondrial oxidative phosphorylation. Conversely, regardless of oxygen conditions, tumor cells prefer anaerobic glycolysis, a less efficient process for obtaining ATP that produces large amounts of lactate. To compensate for this apparent decrease in energy flow, tumor cells increase glucose uptake and trigger alternative pathways to metabolize alternative carbon sources, such as glutamine, and some amino acids, such as arginine and glycine. This change in the energy metabolism confers several advantages to tumor cells, in addition it also provides necessary biomolecules for the high rates of cell division (65, 66).

During the first step of glycolysis, glucose is transformed into glucose-6 phosphate through the phosphorylation of the 6-hydroxyl group of glucose by the enzyme hexokinase (HK). The hexokinase family of enzymes comprises four isoforms (HK1–HK4) (67–69). Isoform 2 (HK2) has been reported to be upregulated in a wide variety of tumors (70–72).

One of the first works that demonstrated the regulation of miRNAs on the HK2 enzyme was published by Fang et al. Interestingly, they demonstrated that miR-125a and miR-143 regulate HK2, which modifies glucose metabolism and cell proliferation in lung cancer cells (73). This finding was confirmed by Peschiaroli et al. in head and neck squamous cell carcinoma (HNSCC)-derived cell lines (74), and by Gregersen et al. in colon cancer cells (75). Another miRNA, miR-199a-5p, regulates HK2 expression and has been reported to be under-expressed in liver cancer cells. Remarkably, overexpression of HIF1α decreased miR-199a-5p expression, which promotes glycolysis and lactate production (30). In stomach cancer cells, miR-181b directly inhibits the expression of HK2 and causes a decrease in glucose uptake and lactate production (76). In addition, miR-155 has also been reported as a regulator of the expression of HK2. Jiang et al. demonstrated that miR-155 regulates the expression of HK2 by two different mechanisms. First, miR-155 promotes the indirect transcription of HK2 through the activation of STAT3, a transcriptional activator of HK2. Second, miR-155 regulates the expression of C/EBPβ, a transcriptional activator of miR-143, whose overexpression is related to the inhibition of HK2 (77).

A couple of works showed that the enzyme responsible for catalyzing the second reaction of glycolysis, glucose-6-phosphate isomerase (GPI), is regulated by miR-200 in breast cancer cells (78) and by miR-302b and miR-17-5p in chicken primordial germ cells (79). Another glycolytic enzyme regulated by miRNAs is phosphofructokinase 1 (PFK1). PFK1 is the main regulatory enzyme for glycolysis; it catalyzes the phosphorylation reaction of fructose-6-phosphate to convert it into fructose-1,6-bisphosphate. In this sense, Yang et al. demonstrated that miR-135 targets PFK1, inhibits aerobic glycolysis, and suppresses tumor growth (31).

Similarly, Aldolase A, a glycolytic enzyme that catalyzes the conversion of fructose-1,6-bisphosphate to glyceraldehyde 3-phosphate (G3P) and dihydroxyacetone phosphate (DHAP), is targeted by several miRNAs. Among the miRNAs that have been reported to regulate Aldolase A expression are the following: miR-122 in liver cells (80), miR-15a and miR-16-1 in leukemia (81), and miR-31 and miR-200a in Y79 retinoblastoma cells (82).

The expression level of glyceraldehyde 3-phosphate dehydrogenase (GAPDH) has been widely used for normalizing quantitative gene expression experiments. GAPDH catalyzes the sixth reaction of glycolysis, where a molecule of NADH is released. Like other enzymes in glycolysis, GAPDH is targeted by some miRNAs such as miR-644a (83) and miR-155 (84).

The last reaction of the glycolysis pathway is catalyzed by pyruvate kinase 2 (PKM2) enzyme. PKM2 dephosphorylates phosphoenolpyruvate to produce pyruvate regardless of oxygen concentration. PKM2 has been reported to be over-expressed in many tumors due to the dysregulation of various miRNAs that down-modulate it. Some of the miRNAs reported to directly regulate the expression of PKM2 are miR-133a, miR-133b, miR-326, and miR-122 (85–87) whereas those that indirectly regulate it are miR-99a, miR-124, miR-137, and miR-340 (88, 89).

## miRNAs Involved in Lactate Metabolism

In tumors, after the glycolysis phase, pyruvate is converted into lactate by the lactate dehydrogenase enzyme (LDH). Some works have reported increased levels of LDH and its correlation with tumor aggressiveness (90–92). Interestingly, LDH expression is also regulated by miRNAs. For instance, miR-375 regulates the subunit B of LDH (LDHB) in maxillary sinus and esophageal anaplasias (93). In addition, subunit A of LHA (LDHA) has been reported to be regulated by miR-34a, miR-34c, miR-369-3p, miR-374, miR4524a/b, miR-323a-3p, miR-200c, miR-30d-5p, and miR-30a-5p in breast cancer cells and osteosarcoma tissues, which induces a decrease in glycolysis, lactate production, ATP generation, and cell proliferation (94–99).

Lactate fluxes are mainly maintained by monocarboxylate transporter (MCTs). MCTs are membrane proteins acting as carriers for lactate, pyruvate, and ketone bodies. Up to now, four MCT isoforms (MCT1, MCT2, MCT2, and MCT4) have been described in humans, and each of them exhibits a distinct cellular distribution (100). In the same way as LDH enzymes, lactate carriers (MCT proteins) are regulated by diverse miRNAs. For example, MCT1 is targeted by miR-29a, miR-29b, miR-124, and miR-495 in pancreatic β cells (101, 102). Another MCT1-regulatory miRNA is miR-342-3p, which promotes alterations in lactate and glucose flows. In addition, miR-342-3p over-expression significantly decreased cell proliferation, viability, and migration in breast cancer cell lines (103). MCT4, another member of the family of lactate transporters, is regulated by miR-145, which causes the accumulation of lactate within tumor cells in hepatocellular carcinoma cells (HCC) (104).

## miRNAs Involved in Glutamine Metabolism

Glutamine metabolism (glutaminolysis) represents the second source of nutrients in cancer cells. Actually, high rates of glutaminolysis are necessary for metabolic reprogramming as it provides substrates for increased lipogenesis and nucleic acid biosynthesis that are critical to preserve the high proliferation rates of tumor cells (105, 106). Glutaminolysis converts glutamine into TCA cycle metabolites through the activity of multiple enzymes. First, glutamine is transported into the cells by solute transporters SLC1A5 and SLC7A5. Once inside the cell, glutamine is converted into glutamate and later into alpha-ketoglutarate (α-KG) by glutaminase (GLS), glutamate dehydrogenase (GDH, and other enzymes, such as glutamate pyruvate transaminase (GPT) for alanine production and glutamate oxaloacetate transaminase (GOT) for aspartate production. In addition, glutaminolysis produces considerable amounts of succinate, fumarate, malate, NADH, and ATP molecules. The transport of glutamine into the cell is strictly regulated by the membrane protein SLC1A5 (also called ASCT2 protein). SLC1A5 and other members of the ASC solute transporters family have been reported to be overexpressed in a wide variety of tumors. Dong J et al. showed that the exogenous expression of miR-137 and miR-122 markedly inhibited the SLC1A5 expression in a dose-dependent manner therefore altering tumor glutamine metabolism (107).

In a well-conducted work, Gao et al. demonstrated that the repression of miR-23a and miR-24b by the oncogenic transcription factor c-Myc resulted in a greater expression of GLS proteins and led to the upregulation of glutamine catabolism (108). Another miRNA reported to regulate GLS protein expression is miR-203, which additionally sensitizes malignant melanoma cells to temozolomide chemotherapy (109). Expression of glutamate cysteine ligase, the rate limiting enzyme of glutathione (GSH) synthesis, is attenuated by miR-18a in liver cancer (110) and by miRNA-153 in glioblastoma (111). Additionally, miR-450a limits the metastatic potential of ovarian cancer cells by targeting a set of mitochondrial mRNAs to reduce glycolysis and glutaminolysis (112).

## miRNAs Regulation of OXPHOS

Oxidative phosphorylation (OXPHOS) is a metabolic pathway combining two cellular processes to generate energy in the form of ATP. First, in an oxidative stage, the electron donors such as NADH and FADH2 are oxidized by the electron transport chain that turns the released energy into a proton gradient across the mitochondrial inner membrane. In the second stage, phosphorylation, ATP synthase uses the proton gradient to phosphorylate ADP to ATP. OXPHOS involves a system of protein complexes with oxidoreductase functions (complex I–IV) and ATP synthase (complex V). Even though OXPHOS is the most efficient way to produce cellular energy, tumor cells prefer to metabolize glucose via aerobic glycolysis. Several studies have recently indicated that, contrary to what is generally accepted, tumor cells could alternate between these two processes, OXPHOS and aerobic glycolysis, depending on the tumor microenvironment (113–115).

Interestingly, it has been proposed that several miRNAs regulate OXPHOS by inducing the inhibition of many components of the electron transport chain. For instance, miR-210 regulates the activity of the mitochondrial complex I (NADH: ubiquinone oxidoreductase) via the iron-sulfur cluster assembly enzyme (ISCU) by reducing the availability of iron and sulfur ions (116). Another study published by Muralimanoharan et al. revealed that miR-210 overexpression significantly reduces the complex III expression of the electron transport chain (ubiquinone:cytochrome c oxidoreductase) (117). Cytochrome c oxidase (complex IV), another enzyme of the electron transport chain, is also regulated by miRNAs. The following miRNAs have been reported to regulate cytochrome c oxidase: miR-181c (118), miR-338 (119), and miR-210 (117).

Finally, ATP synthase (complex V), a transmembrane enzyme that catalyzes ATP synthesis from an ADP molecule, is also regulated by miRNAs. Willers et al. reported that miR-127-5p reduces the expression of the catalytic subunit of ATP synthase (β-F1 subunit) in BT-549 cells in breast cancer (120). Another miRNA, miR-141, reduces the activity of ATP synthase by reducing SLC25A3 proteins (121).

## miRNAs Regulation of Transcription Factors and Signaling Pathways

miRNAs are also capable of modulating metabolic reprogramming through regulating various transcription factors relevant in metabolic pathways (122). The metabolic shift of tumor cells may be a potential strategy to evade programmed cell death and triggers cell survival and growth by activating oncogenes, such as RAS, MYC, and p53 (51, 123–126). Tumor metabolic reprogramming seems to be influenced by oncogenes and tumor suppressor networks. For example, phosphatidylinositol 3-kinase (PI3K), a lipid kinase that regulates the levels of phosphorylated phosphatidylinositol at the plasma membrane and enhances glucose uptake and glycolysis in cancer cell metabolism, is targeted by miR-320, miR-123a, miR-422, miR-506, and miR-136 (127). Catanzaro et al. showed evidence that downregulation of miR-139-5p in pediatric low-grade gliomas drives cell proliferation by regulating PI3K/AKT signaling (128). Furthermore, miR-33a/b, targets metabolic enzymes involved in fatty acid metabolism and the AMPK pathway, whereas miR-29b targets amino acid catabolism, which regulates cancer cell metabolism and biogenesis to support tumor growth and proliferation (61, 129–131). Like PI3K, AKT, and mTORC1, the MYC transcription factor has important metabolic roles beyond enhancing glycolysis. MYC promotes mitochondrial gene expression and mitochondrial biogenesis. MYC mainly depends on glutamine as a carbon source for mitochondrial metabolism (132). The oncogene MYC can bind to the promoter region of other oncogenes such as some miRNAs; for example, miR-9 is frequently upregulated in glioma specimens and cells, and it could significantly enhance proliferation, migration, and invasion of glioma cells (133).

On the other hand, miRNAs regulate important signaling pathways in mitochondria by triggering adaptive mechanisms to optimize the oxidative phosphorylation concerning the substrate supply and energy demands. For example, exogenous exosomes carrying miRNAs can induce metabolic reprogramming by restoring respiration in cancer cells and thus suppressing tumor growth. The exosomal-miRNAs involved in the modulation of cancer metabolism may be used for better diagnoses and therapies (134, 135).

Hypoxia-inducible factor 1 (HIF-1), another pathway related to tumor metabolism, is also regulated by miRNAs. HIF-1 activation can stablished oncogenic signaling by promoting glycolysis of cancer cells; but also, an alternative mechanisms over the glucose carbon mitochondrial metabolism confers HIF-1 a tumor suppressor role in some types of cancer (136, 137). In this way, miR-125-5p, miR-33-5p, and miR-190-5p are known to target the master regulator of oxygen deprivation response, HIF-1 (138). On the other hand, HIF-1 is a key molecule in adapting cancer cells to the reduced oxygen availability in the microenvironment (139–141). HIF-1 induces metabolic reprogramming as it upregulates genes such as HK1, HK2, LDHA, PDK1, GLUT1, and GLUT3, which enhance lactate production through the glycolytic pathway (142, 143). HIF1 also influences the activity of the pentose phosphate pathway, nucleotide biogenesis, angiogenesis, and suppresses the mitochondrial function (144, 145).

**Figure 1:**
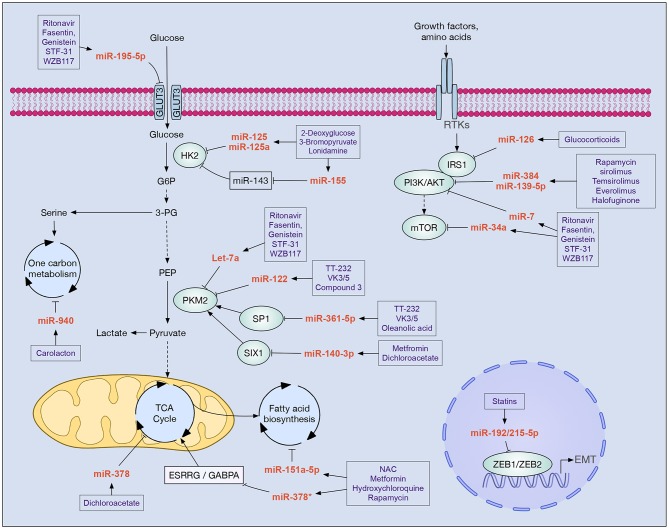
Drugs with clinical potential in cancer that modulate miRNAs implicated in cell metabolism. In boxes are shown drugs that potentially modulate the main miRNAs involved in the metabolic reprogramming of tumor cells. Increased glycolysis flow, alteration of the PI3K/AKT/mTOR pathway, and epithelial-mesenchymal transition (EMT) are key processes that allow tumor cells to reprogram their metabolism in order to survive, proliferate, migrate, and evade new niches. Different miRNAs participate in these processes inhibiting the expression of enzymes (e.g., HK2, PKM2, IRS1, PI3K, AKT, mTOR), transcription factors (e.g., SP1, SIX1, ZEB1, ZEB2, GABPA), and cellular receptors (e.g., GLUT3, ESRRG).

Finally, the oncogene c-MYC regulates HIF1 expression regardless of oxygen levels, and both act in concert to “fine-tune” adaptive responses during tumor growth (146–149). Moreover, it has been reported that more than 50% of tumors have mutations in the tumor suppressor p53, which leads to metabolic changes and contributes to the Warburg effect through the upregulation of c-MYC, HIF-1, and a broad range of genes involved in other aspects of cancer biology, including tumor cell survival and proliferation, migration, drug resistance, and immune evasion (51, 150, 151). The advance in molecular biology techniques has allowed us to detect how a diversity of miRNAs regulate tumors metabolism, as we show in Table 1 and Figure 1.

**Table 1:** Main miRNAs that regulate cellular metabolism in different types of cancer.

**miRNA**	**Location**	**Cancer type**	**Target gene/pathway**	**References**
miR-125a	19q13.41	Hepatocellular carcinoma	HK2	(152, 153)
miR-192/215-5p	11q13.1, 1q41	Colorectal cancer	ZEB1 and ZEB2, Type I collagens	(104)
miR-140-3p	16q22.1	Chronic myeloid leukemia	SIX	(154)
miR-140-3p	16q22.1	Spindle cell oncocytomas	TCA, carbohydrate, lipid metabolism	(155)
miR-940	16p13.3	Glioma	MTHFD2	(156)
miR-139-5p	11q13.4	Pediatric low-grade gliomas	PI3K/AKT signaling	(128)
miR-151a-5p	8q24.3	Malignant pleural mesothelioma	FASN, OXSM, ACACB	(157)
miR-361-5p	Xq21.2	Prostate cancer	Sp1/PKM2 axis	(158)
miR-7, let-7a, miR-34a and miR-143	9q21.32, 9q22.32, 1p36.22; 1p36.22, 5q3	Glioblastoma	Critical regulators of aerobic glycolysis	(159)
miR-125	19q13.41	Hepatocellular carcinoma	HK2	(160)
miR-122	18q21.31	Hepatocellular carcinoma	PKM2 and represses glycolytic metabolism	(161)
miR-126	9q34.3	Mesothelioma, hepatocellular, pancreatic and breast cancer	Insulin receptor substrate-1 (IRS1)	(134)
miR-195-5p	17p13.1	Bladder cancer	GLUT-3	(59)
miR-155	21q21.3	Breast cancer	miR-143	(77)
miR-378	5q32	Breast cancer	ERRγ and GABPA	(162)

## Druggable miRNA-Metabolic Networks With Potential Value for Cancer Therapy

The unveiled connection between cancer profiles and metabolic reprogramming shed light on the reassessment of metabolism-targeting pharmacologic therapies as potential opportunities in cancer. Alterations in key miRNA regulatory networks contribute to the oncogenic transformation of cancer cells through genes involved in the metabolic switch (163). New insights into the altered tumor metabolism have provided novel therapeutic strategies that are being evaluated in preclinical models or clinical trials as effective therapies for many human cancers (164). Pharmacological targeting of altered miRNAs may have therapeutic effects by suppressing relevant cancer signaling pathways without affecting normal cells (165). Furthermore, pleuritic effects of metabolic drugs include miRNAs modulation that impairs signaling pathways and regulates cell energy production, which reveal miRNAs as potential drug targets.

Numerous studies now suggest that drug repurposing, which is the discovery of new therapeutic indications for known drugs, represents an attractive route in drug harnessing in cancer. Unlike the development of new molecules, drug repurposing identifies new uses for existing drugs that already have clinical and safety descriptions (166). Repurposing drugs with an oncological and non-oncological primary purpose, such as metabolic-based drugs, might be an attractive strategy to offer more effective treatment options to cancer patients and faster translate the research knowledge into the clinics (167). Interestingly, a growing body of evidence has shown that many of the antineoplastic effects and improved responses to these metabolic-based drugs may be mediated through induction of tumor suppressor miRNAs and suppression of oncogenic miRNAs.

**Figure 2:**
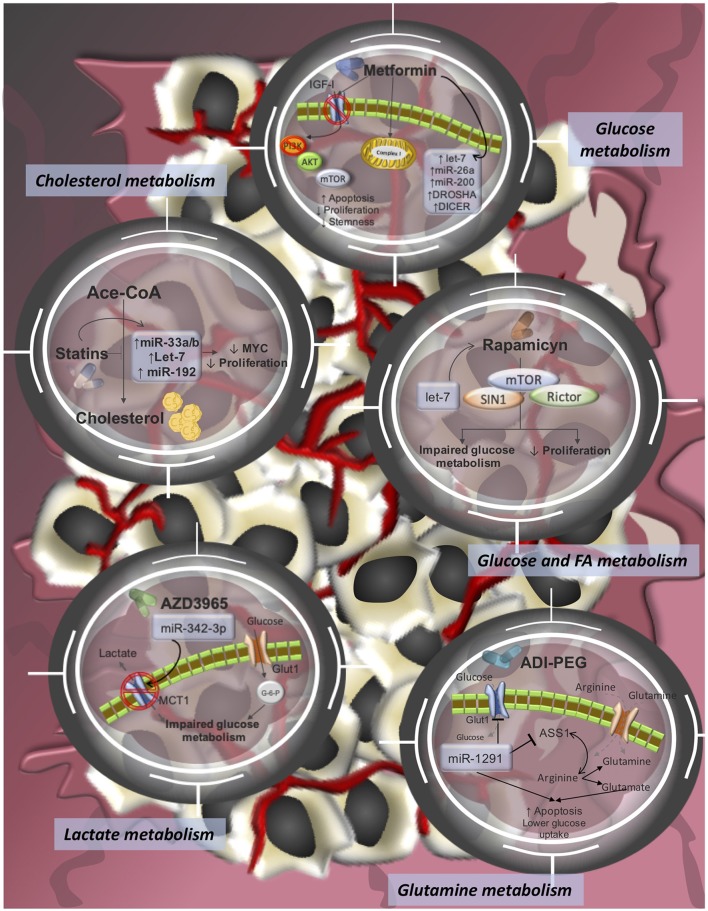
Pharmacological-targeting of tumor metabolism and miRNA-modulating networks of drugs tested in clinical trials or already approved FDA drugs for cancer treatments. It is reported that dysregulations of miRNAs contribute to therapy resistance via drug efflux mechanisms, alterations in drug targets, energy metabolism, Glutamine metabolism, lactate metabolism, cholesterol metabolism, among others.

In this section, we describe existing evidence of molecules with biochemical mechanisms impairing tumor metabolism. These molecules appear as the most promising repurposing and *de-novo* pharmacological interventions as shown by preclinical and clinical studies. Particular emphasis was put on chemo-resistance, which is recognized as a critical cause of treatment failure. It is reported that dysregulations of miRNAs contribute to therapy resistance via drug efflux mechanisms, alterations in drug targets, energy metabolism, DNA repair pathways, evasion of apoptosis, cell cycle control, among others (6, 168, 169). We briefly described below some pharmacologic therapies employed in different metabolic-related diseases and how they could selectively target metabolic pathways in cancer cells and modulate miRNAs networks, we will also comment some of the most relevant evidence of each of the metabolic therapeutically intervention and its anti-carcinogenic properties via miRNA activity. A more extensive over-view of miRNA expression portraits modulated by pharmacological treatment, as well as cooperative or resistance phenotypes toward drug activity is listed in Table 2 and Figure 2.

**Table 2:** miRNAs target by metabolic-drugs or miRNAs related to therapy resistance.

**Drug**	Druggable miRNA/Therapy-resistance miRNA^*^	**Cancer**	**References**
**Targeting glucose metabolism**			
Metformin	↑let-7a, let-7b, miR-26a, 101, 192, 200b and 200c. Over-expression of miR-26a decrease cancer stem-cells markers, an enhanced apoptosis rate. Let-7b re-expression blocks stem cells features	PC BRCA Oral Renal	(170–174)
	↑miR-34a in obese mice reducing its putative targets (Notch, Slug, Snail) ↑miR-34a which in turn restrict Sirt1/Pgc1α/Nrf2 signaling pathway and decrease proliferation rates	PC	(175) (176, 177)
	↓miR-27a which AMPKα and ↑miR-193 family that increased AMPKα and decrease FASN levels, resulting in limiting mammospheres phenotype	BRCA	(178, 179)
	Combined treatment of metformin + FuOx ↓miR-21 and ↑miR-145, that suppress β-catenin and c-Myc signaling expression colon cancer cells	CRC	(180)
	↑miR-141, 200a, 205 and 429 inhibiting EMT, thus, modulating metastatic traits	GC	(181)
	↑mir-124, 182, 27b, let7b and ↓miR-221 and 181a; inhibiting cell proliferation	CLC	(182)
	↑miR-192-5p, 584-3p, and 1246; suppressing cell motility and cell cycle	M	(183)
	↑DROSHA, modulate the miRNA biogenesis, to affect these miRNAs expression	CLC	(182)
	↓miR-222 resulting in enhance abundance of p27, p57, and PTEN ↓miR-222 resulting in enhance abundance of p27, p57, and PTEN	Lung	(184)
	↑DICER expression and miR-33a that targets c-MYC	BRCA	(185)
	↓miR-146a, 100, 425, 193a-3p and 106b involved in cell migration, invasion and proliferation	PCA	(186)
	↑miR-192-5p, miR-584-3p, and miR-1246 enhance EFEMP1 and SCAMP3 downmodulation favoring the suppression of cancer cell motility and growth through G2/M cell cycle arrest and cell apoptosis	M	(183)
	RS:↑miR-21 ↓miR-21 and ↑miR-145 over combined treatment with 5-fluorouracil and oxaliplatin, that suppress β-catenin and c-Myc expression, and consequently reduce cell growth and sphere formation ↓miR-21-5p in cell lines model, xenograft murine model and in tissue from human patients. Since also the pre-miRNA sequence is down-modulated the modulation seems to be at the transcriptional level. Functional reduction of miR-21-5p allow the expression of upstream activators of the AMPK, CAB39L and SESN1	CRCBRCA	(187) (180)(188)
Dichloroacetate (DCA)	Promising therapeutic agents to ↓miR-210	Cancer	(189)
	↑miR-375 resulting in anti-proliferating effects	PCA	(190)
CPI-613	May improve miR-497-5p,−449a,−25-3p,−6838-5p,−520d-3p that down-modulates the expression of Cyclin D3, E1, E2, F, A2, B1 and CDK2 genes of BxPC-3	Cancer	(189)
**Targeting FA metabolism**			
Statins	Lovastatin upregulated miR-33b expression, reduced cell proliferation and impaired c-Myc expression	MB	(191)
	Simvastatin: inhibits the growth of human CRPC cells by suppressing NF-κB and LIN28B and ↑let-7 miRNA family	PCA	(192)
	Simvastatin: ↓miR-34a, which regulates the NAD+-dependent histone deacetylase SIRT1. ↑miR-612, which is known to reduce stemness	BRCA, PCA, OsC	(193)
	Simvastatin is an activator of miR-192 which subsequently led to suppressed proliferation, migration and invasion	CRC	(194)
	Atorvastatin: ↑miR-182 that targets the anti-apoptotic Bcl-2 and p21	PCA	(195)
	↑miR-140-5p activating the transcription factor NRF1 that reduced cell proliferation and induced apoptosis	BRCA	(196)
	Fluvastatin: ↓miR-140-3p-1 and its downstream pathway such as cell growth	BRCA	(197)
	Statin: ↑miR-33a promoting a proliferation inhibitory effect	PCA	(198)
	lovastatin: ↓miR-133a promoting GCH1 important for endothelial nitric oxide synthase	Cancer	(199)
Rapamycin	Rapamycin-dependent miRNA: ↑miR-29b, 21, 24, 221, 106a, and 199a	Renal	(200)
	↑let-7, miR-125a,−125b,−21, and−26a. Rapamycin is mediated by let-7 family with anti-proliferative effects	Renal	(201)
	^*^RS: miR-21 supports mitochondrial function and adaptation to rapamycin	Renal	(200)
	Long-term rapamycin treatment RS: ↑MYC that results in ↑miR-17–92	Brain	(201)
Aspirin and non-steroidal anti-inflammatory agents	↑miR-98 that targets WNT1, suppressing cell proliferation	Lung	(202)
	Sulindac drug: ↓miR-9,−10b,−17, and−21 by suppressing NF-κB-mediated transcription of miRNAs	BRCA CRC	(203)
	↓miR-21 decreasing cell proliferation and invasion upon inactivation of β-catenin/TCF4 signaling	CRC	(204)
	↑let-7 by decreasing the miRNA-sponge H19, resulting in the down-modulation Hypoxia-inducible factor 1α reducing l PDK1, attenuating glycolysis	BRCA	(195)
	Celecoxib: ↑miR-29c supress the oncogen MCL-1 reducing apoptosis	GC	(205)
TVB-2640	miR-15 and miR-16-6: Inhibition of FASN: Agonist effect	BRCA	(206)
**Targeting lactate metabolism: LDHA inhibitors**			
AZD3965	miR-342-3p: Inhibition of the monocarboxylate transporter MCT1: Agonist effect	BRCA	(103)
**Antimetabolite chemotherapeutic agents**			
Methotrexate (MTX)	^*^RS: ↑*miR-24 SNP*results		(207)
	^*^RS: ↑miR-140	OsC, CRC	(208)
	^*^RS: ↑miR-215 modulated DTL, a cell cycle-regulated gene	OsC, CRC	(209)
Capecitabine	↑miR-125b-5p ↑miR-137	Cancer	(189)
5-Fluorouracil	↓Relevant oncogenes such as miR-210	HCC CRCOsC	(208, 210, 211)
	↑Relevant tumor suppressor miRs: let-7 family, miR-15b,−16,−23a,−23b, and−200c	BRCA	(189)
	^*^ES: ↑miR122 through the inhibition of M2 splice isoform of pyruvate kinase (PKM2) *in vitro* and *in vivo*	CRC	(212)
	^*^RS: ↑miR-21 and−221	BRCA	(213)
	^*^RS: ↑miR-21,−34,−140	HCC CRCOsC	(212)
Gemcitabine	May impact the expression of 56 relevant miRNAs such as miR-200,−205,−27a,−27b, and let 7 family	Cancer	(214, 215)
	^*^ES: ↑microRNA-218 by inhibiting the secretion of HMGB1 by PANC-1 cells and the PI3K/Akt pathway	PC	(212)
	^*^RS: ↑miR-21,−34,−140	PC	(214, 215)
**Targeting glutamine metabolism**			
Pegylated arginine deiminase (ADI-PEG)	Bioengineered pre-miR-1291 processed to high levels of mature miR-1291. ^*^ER: ↑miR-1291 increases sensitivity to ADI-PEG (trough modulation of ASS1 and GLUT1)	PC	(216)

* *Therapy-resistance miRNA. ↑, over-expression; ↓, down-regulation. Therapy-resistance miRNA: RS, reduce sensitivity; ES, enhanced sensitivity. Cancer: BRCA, breast cancer; CRC, colorectal cancer; PCA, prostate cancer; PC, pancreatic cancer; HCC hepatacarcinoma; CLC, cholangiocarcinoma; MB, medulloblastoma; OsC, osteosarcoma; GC, Gastric; M,Melanoma*.

## Targeting Glucose Metabolism: Metformin

Metformin, a commonly prescribed drug for treating type 2 diabetes, inhibits the mitochondrial complex I that impairs respiration, which results in a systemic impede of glucose uptake and neoglucogenesis (217–220) that reduces blood glycemia and insulinemia in hyperglycemic/diabetic patients. The tumor-suppressing effect of metformin has been reported in epidemiological studies describing a statistical association between metformin use and improved clinical outcomes in cancer (221–224). One striking example of this onco-suppressive feature is the cooperative effect of metformin and neoadjuvant chemotherapy to achieve complete tumor regression in some breast cancer patients (225). Although the precise anti-tumorigenic mechanism of action is not well-described, recent studies have shown that metformin can partially direct mitochondrial complex I inhibition, reduce NADH oxidation, and increase AMP/ATP ratio in tumors, with the consequent inhibition of mTOR signaling and decrease of fatty acid and cholesterol synthesis (218, 220, 226). Thus, metformin favors a catabolic process over an anabolic one in tumor cells. Overall, this metabolic pressure causes proliferation decline and triggers apoptosis in cancer cell lines [(227); Table 2 and Figure 2].

A variety of evidence, both *in-vitro* and *in-vivo* along with epidemiological studies, supported the protective effect of metformin against cancer development (228–231). Even more, the role of metformin on cancer not only fall in limiting its incidence, but also as a novel therapeutically intervention as shown by the 335 registered clinical trials that have evaluated patients benefit of incorporate Metformin in their treatment. The underlying mechanism of the anticancer activity of Metformin can be partially explained through its ability to modulate miRNA expression, activity and biogenesis in a variety of tumor types (Table 2 and Figure 2). For instance, overexpression of the tumor suppressors let-7, miR-26, and miR-200 family members has been reported in the literature as a pleuritic effect of Metformin molecular activity in breast, colorectal, pancreatic, oral and renal cancer. Briefly, Metformin up-modulates let-7a, that epigenetically inhibits the oncomiR miRNA-181a, which actively participated in the epithelial-to-mesenchymal transition, thus, abrogating this aggressive phenotype in BRCA (170). In CRC, the metabolic drug overexpress let-7, miR-200b/c, and miR-26a that limit the stem-like phenotype, which has been linked to poor clinical outcomes (171). Consistently, in pancreatic tumors Metformin induces the expression of miR-26a and let-7c miRNAs reducing cell proliferation, invasion, and migration. Particularly, miR-26a down-regulates the oncogene HMGA1 contributing to the observed phenotype (172). Studies in oral cancer cell models reveal that Metformin significantly increases miR-26a levels which directly decreases Mcl-1 expression that enhances apoptotic rates and reduces tumor-cell viability (173). Finally, in renal carcinoma Metformin treatment limits cell proliferation by miR-26a up-modulation that in turn down-regulates Bcl-2, cyclin D1 and upregulates the tumor suppressor PTEN, which all together influence cell cycle and cell death (174).

## Targeting Aerobic Glycolysis: PDK Inhibitors

Dichloroacetate (DCA, PDK inhibitor) is a small molecule that inhibits the pyruvate dehydrogenase kinase (PDK) and regulates mitochondrial pyruvate dehydrogenase complex that catalyzes the irreversible decarboxylation of pyruvate into acetyl-CoA (232). PDK is overexpressed in several tumors and favors pyruvate conversion into lactate (233). Inhibition of PDK by DCA in cancer cells prompts glucose oxidation, reverses mitochondrial apoptosis, and suppresses tumor growth (234). CPI-613 is a novel anticancer agent (lipoic acid analog) that inhibits PDK through targeting lipoyl-binding pockets and selectively target the altered mitochondrial energy metabolism in tumor cells and produces changes in mitochondrial and redox status, which leads to tumor cells death (232, 235, 236). One of the main clinical challenges in colorectal cancer management is the development of chemoresistance. Interestingly, DCA treatment improve chemosensitivity to 5-fluorouracil. The evidence pointed out that the DCA over-express miR-149-3p which consequently enhanced 5-FU-induced apoptosis. Importantly, miR-149-3p is a post-transcriptional regulator of PDK2 transcript. Thus, DCA treatment overcome chemoresistant phenotype by modulating miR-149-3p/PDK2 axis (237).

## Targeting FA Metabolism

Several pieces of evidence propose that targeting *de novo* fatty acid synthesis might be effective in the treatment of some cancers. For example, statins, cholesterol-lowering drugs, have been recently related to antitumor, cytostatic, and cytotoxic activity in diverse clinical trials of advanced malignancies (238); however, the studies are still inconclusive. Epidemiological studies have shown that statins lower the risk of presenting lung, breast, bowel, and prostate cancer (239, 240). Furthermore, different preclinical *in-vitro* studies show that statins may produce a variety of antineoplastic responses in cancer cells, including a cytostatic effect (cell cycle G1/S phase arrest), pro-apoptotic activity by downmodulating BCL-2 (241, 242), anti-metastatic properties through NF-kB and matrix metalloproteinase inactivation (243, 244) and anti-angiogenic properties. Different studies have provided novel evidence of the pleiotropic effects of statins independent to its cholesterol signaling modulation in cancer. For instance, *in-vitro* assays have shown that more than 400 miRNAs are altered by statins interventions. Including, some well-known tumor suppressor miRNAs such as miR-612, which is up-modulated after statins treatment promoting cancer cell differentiation and enhancing cancer cells response to chemotherapy (193). In another study, miR33a (198), and miR-33b (191) resulted up-modulated and participates in the anti-oncogenic properties of statins by promoting proliferation inhibitory effects and down-regulating the oncogene c-Myc. Another statin-regulated miRNA is miR-182, which down-regulates the antiapoptotic Bcl-2 transcript and consequently favors cell apoptosis (195). In a more complex regulatory circuit, simvastatin reduces NF-κB and LIN28B expression and subsequently increased let-7 levels, that in summary significantly inhibited cell viability and clonal proliferation [(192); Table 2 and Figure 2].

In a different fashion, rapalogs that inhibit mTOR (e.g., rapamycin and its derivatives, everolimus, and temsirolimus) exhibit anti-tumor effects by targeting PI3K/Akt/mTOR axis and cell proliferation. A wide spectrum of tumors is being evaluated in monotherapy or in combination. Temsirolimus and everolimus have been recently approved for the treatment of patients with advanced renal cell carcinoma (245, 246). Since mTOR is also involved in glucose metabolism by stimulating GLUT1, it is reasonable to propose a combinatory therapy with metformin to synergistically kill tumor cells [(247); Table 2]. Once again, let-7 family is one of the most reported miRNAs related with Rapamycin mechanism, playing a dual role. In one hand, in short-term treatments the inhibitory effect of rapamycin over cancer cells is mediated by increased expression of let-7 members that regulates c-MYC post-transcriptionally regulates c-MYC. On the other hand, re-expression of let-7 restore rapamycin sensitivity in resistant tumor cells (201). Long-term rapamycin treatment up-modulates miR-17–92 cluster that is related to rapamycin resistance, probably by its positive regulation over c-Myc (201). From a combinatory point of view rapamycin and metformin are able to synergize their activities against cancer cells, since this last one inhibits miR-21-5p which induces signaling of mTOR, a rapamycin-target (188).

Finally, TVB- 2640 compound is one of the first bioavailable fatty acid synthase (FASN) inhibitor to enter clinical trials for breast, colon, and astrocytic tumors, in combination with chemotherapy with the aim of enhancing clinical responses and prolonging stable disease times (NIH). Its antineoplastic activity leads to reduced cell signaling, induces tumor cell apoptosis, and inhibits cell proliferation in tumor cells by restricting lipid signaling, mainly fatty acid production, which is necessary to satisfy tumors metabolic needs [(248–250); Table 2 and Figure 2].

## Aspirin: Anti-inflammatory and Metabolic Drug in Cancer Cells

Aspirin, a non-steroidal anti-inflammatory drug (NSAIDs), has shown metabolic and antitumor properties (251). Aspirin may impair tumor cell migration and metastasis through preventing platelet clot formation (252). Aspirin also activates AMPK and inhibits mTOR and FA synthesis in cancer cell lines (253). Recently, aspirin has been demonstrated to have effective anti-tumor effects against RAS/RAF-mutant cells in colorectal cancer by simultaneously affecting BRAF/CRAF dimerization and hyper-activating the AMPK and ERK pathway [(254); Table 2 and Figure 2]. Besides the well-described cardioprotective effects of NSAIDs, there are substantial preclinical, clinical, and observational data that supports its activity in preventing cancer, with strong evidence in colorectal (255), lung (256), and ovarian cancer (257, 258). In preclinical studies NSAIDs administration confer a chemopreventive effect in different cancer cell models and *in-vivo* assays, probably via miRNA modulation. Recently, a novel mechanism of action of aspirin has been reported, in which the drug induces the expression of well-known tumor suppressors miRNAs, such as miR-98 that in turn suppress WNT1 and consequently limits cell proliferation in lung cancer (202). Moreover, NSAIDs favor let-7 expression by decreasing the abundance of one of its ncRNA-sponge, attenuating in this way glycolysis in breast cancer [(195); Table 2]. Anti-inflammatory drugs are also able to abrogate the oncogene miR-21, that results in low cell proliferation and invasion rates in BRCA and CRC (203, 204).

## Targeting Lactate Metabolism: LDHA Inhibitors

Several clinical trials evaluating LDHA inhibitors in different solid cancers are currently underway. One mechanism of action of LDHA inhibitors is to limit lactate export from cancer cells into the extracellular space. Accumulating intracellular lactate moves LDHA catalyzed-reaction to produce pyruvate, which prevents NAD+ regeneration and affects the energy source that established a fine competition between cancer cells that resulted in cell death. AZD3965, a drug affecting lactate metabolism, inhibits lactate transporter MCT1, which is overexpressed in several tumors and is associated with poor outcomes (259–262). MCT1 inhibitors probably synergize with the exogenous restauration of miR-342-3p that should provide a more effective inhibition of lactate transportation, which result in loss of cancer cell metabolism homeostasis [(103); Table 2 and Figure 2].

## Anti-tumoral Therapy With Antimetabolite Chemotherapeutic Agents

Antimetabolites as chemotherapeutic agents (e.g., methotrexate, capecitabine, 5-fluorouracil, and gemcitabine) are small molecules that resemble nucleotide metabolites; they inhibit the activity of enzymes involved in nucleotide synthesis by preventing cell division and triggering cell death. They are widely used in clinics to treat cancer since neoplastic cells have an increased metabolic demand that requires a huge nucleotide biosynthesis and DNA replication (263). More in detail, methotrexate is a folate analog that inhibits carbon transfer reactions required for *de novo* nucleotide synthesis. Fluorouracil (5-FU) is a synthetic analog of uracil that inhibits thymidylate synthase by limiting the availability of thymidine nucleotides for DNA synthesis (264) and has been reported that enhances the expression of relevant tumor suppressors such as let-7 family, miR-15b,−16,−23a,−23b, and−200c, some of them well-describe metabolic modulators (189). Moreover, 5-FU represses miR-210 (208, 210, 211), that down-modulates GPD1L, a negative regulator of HIF, restricting HIF-1α stability [(265, 266); Table 2]. Similarly, capecitabine is widely used in chemotherapies for gastrointestinal cancers. It halts tumor cells by inhibiting DNA and RNA synthesis and limiting the precursor of thymidine triphosphate (267, 268). Gemcitabine, another nucleoside analog, is intercalated into the DNA molecule and blocks DNA polymerases (269). Notably, the literature reports its effect over several miRNAs such as miR-200,−205,−27a,−27b, and let 7 family [(215, 269); Table 2]. All these agents can achieve important clinical responses and lead to complete remission in many cases.

In recent years, there has been substantial attention to the role of miRNAs in regulating metabolic reprogramming. Researchers have tried to reveal the mechanisms that regulate metabolic alterations in tumor cells and identify the interactions (miRNA-mRNA, miRNA-transcription factor, and miRNA-metabolic pathway) that are susceptible of being therapeutically actionable. Although studies are still incipient, robust data have been generated, describing how miRNAs directly or indirectly regulate the dysregulated metabolism of tumor cells. Based on the evidence described in this work, it is appropriate to hypothesize that there are miRNA interactions susceptible of being modulated by therapeutic interventions to reverse the metabolic alterations that allow tumor cells to uncontrollably proliferate. In addition, it is necessary to emphasize the usefulness of miRNAs-based gene therapies to enhance the regulatory activity of those identified miRNAs. However, more studies need to be conducted in a broader spectrum of components of the energetic metabolism of tumor cells, such as enzymes, transcription factors, positive regulators, and enhancers to provide more evidence on the impact of regulation mediated by miRNAs and their signaling networks on oncogenic processes.

## New Druggable Targets With Huge Impact in Cancer Metabolism: the Emergence of miRNA-Based Therapies

In the last section we discussed how metabolic-target drugs and chemotherapy can modulate miRNA signaling programs as a beneficial pleiotropic effect. But it is also necessary to emphasize the usefulness of miRNAs-based therapies to improve or moderate their regulatory activity. Recent advances have permitted to study the effects of directly manipulating cellular miRNA levels by suppressing the expression of oncomiRs, that somehow enhance cancer metabolism, and which are frequently overexpressed in human cancers. Or on the contrary, by reestablishing the expression of tumor suppressor miRNAs that in many cases collaborate to restrict cancer energetics programs (270). Evidences obtained from these studies have prompted the designing and refinement of dedicated technology aimed at, either, inhibiting miRNAs (i.e., antisense oligonucleotides, locked nucleic acid, antagomiRs, miRNA sponges, and small molecule inhibitors that inactivate mature miRNA sequence in the RISC complex) (271–273) or restoring their levels by mimic sequences that can be recognized by Dicer and Ago2 proteins to be functional. Notable, miRNA delivery systems have been improved during the last years, resulting in robust and more specific devices such as liposomes, adenovirus, adeno-associated virus, EDV nanocell, and nano-particles accompanied with conjugate antibodies (274–276).

Below we briefly describe some examples of clinical trials that have been evaluated the therapeutic impact of targeting miRNAs involved in the regulation of emerging hallmarks of cancer like tumor metabolism, already described in previous sections. For instance, MRX34 was the first miRNA-based therapy undergoing in a clinical trial for cancer treatment, its aim was to re-express miR-34, that regulates LDHA, by introducing a mimic sequence through the lipid carrier NOV40 to treat patients with lymphoma, melanoma, multiple myeloma, liver, small cell lung, and renal carcinoma. Unfortunately, although promising results were observed, the trial was terminated due to severe immune-related reactions developed by some patients (277).

The first completed phase 1 trial evaluated the TargomiR technology, intended for delivering miRNA mimic sequences in vehicles containing bacterially derived minicells and a targeting moiety antibody against EGFR to treat non-small cell lung cancer. A similar example is the MesomiR-1 drug, which reintroduces miR-16, a miRNA that regulates Aldolase A in glycolysis process (278, 279). Another, drug delivery system being evaluated in stage 1 clinical trial is the locked oligonucleotide acid-modified inhibitor for miR-155 (MRG-106), as part of the clinical intervention for cutaneous T-cell lymphoma patients (280, 281). This therapeutic intervention re-expresses miR-155 targets such as miR-143, that negatively regulates HK2 and consequently limits the active glycolytic phenotype (77). Other examples include the new miRNA delivery system from Regulus company named RGLS5579, an anti-miRNA against miR-10b, for patients diagnosed with glioblastoma multiforme (282). Interestingly, under hypoxic conditions, HIF1 upregulates the transcription factor TWIST that results in the induction of the oncomiR miR-10b (283).

A further candidate of miRNA-based therapeutic currently under evaluation by Regulus and Sanofi companies, although not for cancer patient's treatment, is RG-012 which silences miR-21 in patients with Alport syndrome (284). Along the text we widely discussed miR-21 activity as a promoter of the tumoral-metabolism and its role in resistance against metabolic-based drugs. Miragen, another company, maintains also an active phase 1 study for miR-29 mimic (MRG-201) to treat keloid, fibrosis and scar tissue formation (ClinicalTrials: NCT03601052). Importantly, miR-29 is frequently lost in cancer and has been reported to negatively regulates MCT1, a lactate transporter (101, 102).

Lastly, combinatorial therapy strategies have provided successful results to treat cancer since this approach can target several tumor cell survival pathways and establish molecular landscapes to overcome resistance, offering a holistic way to reduce tumor development and evolution (285). Taking advantage of the technological advances, chemotherapeutic agents can be coordinately administrated with miRNA-based therapeutics to provide synergistic effects and enhance patient response. Since, these examples represent the first generation of miRNA-based therapeutics, there are some challenges and limitations. As an illustration, preclinical experiments in *in-vivo* models have shown low RNA stability, numerous mRNAs targets can be regulated by a single miRNA and different biological effects can be achieved by a miRNA in different tissues (286). Thus, it is important to guarantee tumor-specific delivery and local retention of miRNAs, for example by nanoparticle which facilitates target-specific shipment of miRNAs (286, 287).

## Perspectives: How to Take Advantage of the Local and Systemic Metabolic Context and its Connection to microRNA Regulatory Circuits in Cancer?

In addition to tumoral-intracellular metabolic reprogramming, tumor cells encounter a variety of systemic factors that can influence tumorigenesis and cell metabolism (27, 34, 38, 41, 164, 288). For instance, obesity is a metabolic disorder that promote tumor growth and a connection between obesity and certain cancers, including colorectal, renal, breast cancer, esophageal, adenocarcinoma, thyroid, endometrial, prostate, and leukemia, have been reported in numerous cohort studies (289–292). In recent years, there has been substantial attention to miRNA roles in obesity-linked cancer (293). miRNA regulation programs can modulate adipogenic differentiation by controlling signaling pathways related to its biogenesis, additionally, several miRNAs associated with obesity also have well-described roles in carcinogenesis, thus, their deregulated expression portrait may act as a functional link between obesity and cancer (294–296).

Furthermore, over the last decade, a huge advent of next-generation sequencing occurred, allowing to deeply characterize the diversity of microorganisms that colonize human epitheliums, known as microbiota. Human microbiome produces small molecules and metabolites through a complex community network with relevant biological effects both at local and systemic levels and its dysregulation contributes to cancer establishment, progression and therapy response (297–300). Carcinogenesis is a complex process on which exogenous, as well as, endogenous factors could impact in different ways on malignant transformation. Among endogenous factors, metabolites generated as byproducts of metabolic activity can either act as carcinogen compounds (i.e., nitrosamines, conversion of alcohol to acetaldehyde, and tumor-promoting secondary bile acids) or as anticarcinogens (i.e., activation of dietary phytochemicals and inactivation of hormones that stimulate tumor cells growth). Even more, metabolism of different substances within the body can be affected by different health conditions like diabetes or obesity, which is characterized by chronic inflammation. In this context, bacterial metagenome has revealed to be an important player in fine-tuning tumor metabolic function, as is enriched in genes that participates in nutrients, bile acid and xenobiotic metabolism, as well as biosynthesis of vitamins and isoprenoids, therefore has become an emergent factor that affects tumor development (301–303). Based on these novel data, the gut microbiome is increasingly being recognized as a dynamic ecosystem influenced by environmental conditions such as diet and drug therapy with relevant effects on tumoral biology and metabolism (304, 305). As an open system, gut microbes elicit their effects on cancer cells via their capacity to induce pro-inflammatory responses (306–308) or more indirectly by the production of secondary metabolites (309–311). Recent evidence showed that short chain fatty acids (SCFAs), hydrogen sulfide (H2S), bile acids, and some other metabolites are produced by gut microbiota and impact the genome and epigenome of cancer cells, including miRNAs. Thus, the gut microbiome is an important regulator of host transcriptional dynamics in part through the establishment of inter-communications via miRNA signaling (312).

Host microbiome has pointed out as a potential modulator of cancer metabolism and could be a future target for precision medicine. While there is less evidence of how microbiota affects most of the miRNA landscapes in human tumors, there are growing data that explain how the microbiota confers some effect on cancer pathways in colorectal cancer (CRC). Under physiological conditions, the microbiota promotes a metabolic niche that produces a huge amount of the energy required by the intestinal epithelial cells (313) through the production of butyrate, a SCFAs, as a result of complex carbohydrates fermentation. CRC cells preferentially use glucose over butyrate as the major source of energy, resulting in a gut microbiome related dysbiosis (314). In the tumoral context, low butyrate concentrations enhance MYC expression, which in turn up-modulates the levels of the oncogenic miR-17-92 cluster (315). The overexpression of miR-17-92a cluster has been shown to enhance cell proliferation, metastasis, and angiogenesis (316–318). This data demonstrates an antitumor mechanism of butyrate over the MYC /miR-17-92a axis in CRC cells. As exemplified, miRNAs activity is a relevant feature in mediating metabolic changes and modulating the interaction of host transcriptional portrait and microbiota. Some other evidences are described in Table 3. Results from numerous studies now suggest an additionally level in the complex interplay between miRNAs and gut microbiome, including data describing the influence of miRNAs in controlling gut composition and growth rates by improving selectively pressure on the surrounding microenvironment (Table 3).

**Table 3:** miRNA portrait and gut microbiota in cancer.

**miRNA**	**Activity**	**Cancer**	**References**
miR-182, miR-503, and mir-17~92 cluster	Differentially expression of these oncogenic miRNAs was correlated with the relative abundances of: *Firmicutes, Bacteroidetes*, and *Proteobacteria*. Possible role of these miRNAs in driven glycan production in tumor location through the recruitment of pathogenic microbial taxa and thus impact tumor development	CCR	(319)
Upregulation of miR-21	*Fusobacterium nucleatum* induces CRC cell proliferation by up-modulating the oncogenic miR-21 via TLR4 signaling	CRC	(320)
Upregulation of miR-20a-5p	The colibactin genotoxin produced by *Escherichia coli* promotes cellular senescence by the upregulation of miR-20a-5p, which in turn downregulates SENP1, resulting in the proliferation of uninfected cells and, subsequently, tumor growth. The over-expression of miR-20a-5p also alters p53 SUMOylation, which has been shown to promote tumor growth and metastasis	CRC	(321–323)

**Figure 3:**
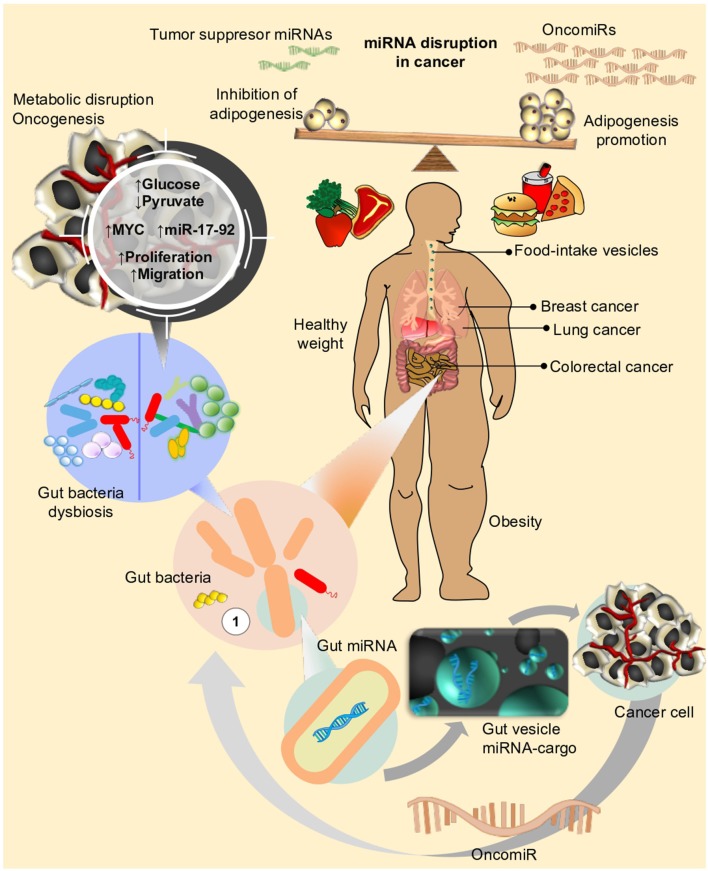
Life style and diet has an impact on different metabolic mechanisms in human cells. Disruption of metabolic fluxes, might particularly affect expression of genes and miRNAs related to control of cell proliferation, cell cycle, and adhesion, eventually leading or favoring neoplastic processes to take place in different organs (i.e., Breast, Prostate, Lung, Colon, etc.). Microbiota, on the other side, the new star player in the complex interaction between environment and human organism, can also influence the effect of nutrients or drug intake within host. In an unhealthy weight scenario (i.e., obesity), disequilibrium in adipogenesis leads to chronic inflammation and triggering of signals for over-expression of oncomiRs. Under this condition, dysbiosis (e.g., loss of balance in gut bacteria composition) could further concur to sustain or even enhance the metabolic perturbations favoring neoplastic transformations.

Furthermore, results from numerous studies suggest that intestinal miRNAs come from two main sources: host and food (55, 324). The intestinal epithelial cells are the main contributors of host-derived miRNAs, but miRNAs contained in food can as well be absorbed by the host and regulate gene expression in a cross-species regulation manner (325, 326). Recently it has been showed that Ginger derived exosome-like vesicles, containing RNA, are taken up by the gut microbiota and can alter microbiome composition and host physiology. Briefly, the exosomal particles are preferentially engulfed by *Lactobacillus rhamnosus* and the exosomal microRNAs-cargo target various genes in the bacteria, such as Ginger miR7267-3p that mediate the production of IL-22, favoring an improvement in the colitis via IL-22-dependent mechanisms (324). These findings reveal how plant products and their effects on the microbiome may be exploited to specially target host processes to modify tumor growth through specific diet interventions (Figure 3).

Although studies are still incipient, robust data have been generated, describing how microRNAs serve as important communication factors between the gut microbiome and the host. On the basis of these evidences, it's appropriate to hypothesize there is an open bi-directional communication between host cells and microbes, potentially mediated through miRNA activity. However, more studies are required to be conducted in a broader spectra of cancers, to provide more evidence on the impact of gut microbiota and their miRNAs signaling networks on oncogenic and metabolic processes (300), which finally will allow us take advantage of these changes and devise new strategies to translate the modulation of metabolic alterations into patient management.

## Author Contributions

AP-T, GH, SR-C, MJ-G, IS-G, RR-B, and SM conceived, designed, and wrote most of the manuscript. RM-M, CA-C, VF-O, RÁ-G, and LH contributed to the writing as well as gathered information for the manuscript. SR-C, MJ-G, IS-G, and RR-B assembled figures and tables.

### Conflict of Interest

The authors declare that the research was conducted in the absence of any commercial or financial relationships that could be construed as a potential conflict of interest.

## References

[1] Lee RC Feinbaum RL Ambros V. The *C. elegans* heterochronic gene lin-4 encodes small RNAs with antisense complementarity to lin-14. Cell. (1993) 75:843–54. 10.1016/0092-8674(93)90529-y 8252621

[2] Wightman B Ha I Ruvkun G. Posttranscriptional regulation of the heterochronic gene lin-14 by lin-4 mediates temporal pattern formation in *C. elegans*. Cell. (1993) 75:855–62. 10.1016/0092-8674(93)90530-4 8252622

[3] Behm-Ansmant I Rehwinkel J Doerks T Stark A Bork P Izaurralde E. mRNA degradation by miRNAs and GW182 requires both CCR4:NOT deadenylase and DCP1:DCP2 decapping complexes. Genes Dev. (2006) 20:1885–98. 10.1101/gad.1424106 16815998 PMC1522082

[4] Behm-Ansmant I Rehwinkel J Izaurralde E MicroRNAs silence gene expression by repressing protein expression and/or by promoting mRNA decay. Cold Spring Harb Symp Quant Biol. (2006) 71:523–30. 10.1101/sqb.2006.71.013 17381335

[5] Vella MC Choi E-Y Lin S-Y Reinert K Slack FJ. The *C. elegans* microRNA let-7 binds to imperfect let-7 complementary sites from the lin-41 3'UTR. Genes Dev. (2004) 18:132–7. 10.1101/gad.1165404 14729570 PMC324419

[6] Garofalo M Croce CM. microRNAs: master regulators as potential therapeutics in cancer. Annu Rev Pharmacol Toxicol. (2011) 51:25–43. 10.1146/annurev-pharmtox-010510-100517 20809797

[7] Kim VN Han J Siomi MC. Biogenesis of small RNAs in animals. Nat Rev Mol Cell Biol. (2009) 10:126–39. 10.1038/nrm2632 19165215

[8] Bartel DP. MicroRNAs: genomics, biogenesis, mechanism, and function. Cell. (2004) 116:281–97. 10.1016/s0092-8674(04)00045-5 14744438

[9] Ha M Kim VN. Regulation of microRNA biogenesis. Nat Rev Mol Cell Biol. (2014) 15:509–24. 10.1038/nrm3838 25027649

[10] Lee Y Kim M Han J Yeom K-H Lee S Baek SH. MicroRNA genes are transcribed by RNA polymerase II. EMBO J. (2004) 23:4051–60. 10.1038/sj.emboj.7600385 15372072 PMC524334

[11] Han J Lee Y Yeom K-H Kim Y-K Jin H Kim VN. The Drosha-DGCR8 complex in primary microRNA processing. Genes Dev. (2004) 18:3016–27. 10.1101/gad.1262504 15574589 PMC535913

[12] Wang Y Medvid R Melton C Jaenisch R Blelloch R. DGCR8 is essential for microRNA biogenesis and silencing of embryonic stem cell self-renewal. Nat Genet. (2007) 39:380–5. 10.1038/ng1969 17259983 PMC3008549

[13] Yi R Qin Y Macara IG Cullen BR. Exportin-5 mediates the nuclear export of pre-microRNAs and short hairpin RNAs. Genes Dev. (2003) 17:3011–6. 10.1101/gad.1158803 14681208 PMC305252

[14] Zhang H Kolb FA Jaskiewicz L Westhof E Filipowicz W. Single processing center models for human Dicer and bacterial RNase III. Cell. (2004) 118:57–68. 10.1016/j.cell.2004.06.017 15242644

[15] Koscianska E Starega-Roslan J Krzyzosiak WJ. The role of Dicer protein partners in the processing of microRNA precursors. PLoS ONE. (2011) 6:e28548. 10.1371/journal.pone.0028548 22163034 PMC3232248

[16] Fareh M Yeom K-H Haagsma AC Chauhan S Heo I Joo C. TRBP ensures efficient Dicer processing of precursor microRNA in RNA-crowded environments. Nat Commun. (2016) 7:13694. 10.1038/ncomms13694 27934859 PMC5155159

[17] Chendrimada TP Gregory RI Kumaraswamy E Norman J Cooch N Nishikura K. TRBP recruits the Dicer complex to Ago2 for microRNA processing and gene silencing. Nature. (2005) 436:740–4. 10.1038/nature03868 15973356 PMC2944926

[18] Meister G Landthaler M Patkaniowska A Dorsett Y Teng G Tuschl T. Human Argonaute2 mediates RNA cleavage targeted by miRNAs and siRNAs. Mol Cell. (2004) 15:185–97. 10.1016/j.molcel.2004.07.007 15260970

[19] Friedman RC Farh KK-H Burge CB Bartel DP. Most mammalian mRNAs are conserved targets of microRNAs. Genome Res. (2009) 19:92–105. 10.1101/gr.082701.108 18955434 PMC2612969

[20] Roccaro AM Sacco A Thompson B Leleu X Azab AK Azab F. MicroRNAs 15a and 16 regulate tumor proliferation in multiple myeloma. Blood. (2009) 113:6669–80. 10.1182/blood-2009-01-198408 19401561 PMC2710922

[21] Chen J-F Tao Y Li J Deng Z Yan Z Xiao X. microRNA-1 and microRNA-206 regulate skeletal muscle satellite cell proliferation and differentiation by repressing Pax7. J Cell Biol. (2010) 190:867–79. 10.1083/jcb.200911036 20819939 PMC2935565

[22] Yamakuchi M Ferlito M Lowenstein CJ. miR-34a repression of SIRT1 regulates apoptosis. Proc Natl Acad Sci USA. (2008) 105:13421–6. 10.1073/pnas.0801613105 18755897 PMC2533205

[23] Schickel R Park S-M Murmann AE Peter ME. miR-200c regulates induction of apoptosis through CD95 by targeting FAP-1. Mol Cell. (2010) 38:908–15. 10.1016/j.molcel.2010.05.018 20620960 PMC2904349

[24] Otsubo T Akiyama Y Hashimoto Y Shimada S Goto K Yuasa Y. MicroRNA-126 inhibits SOX2 expression and contributes to gastric carcinogenesis. PLoS ONE. (2011) 6:e16617. 10.1371/journal.pone.0016617 21304604 PMC3029394

[25] Hu Q Du K Mao X Ning S. miR-197 is downregulated in cervical carcinogenesis and suppresses cell proliferation and invasion through targeting forkhead box M1. Oncol Lett. (2018) 15:10063–9. 10.3892/ol.2018.8565 29928375 PMC6004723

[26] Lu H Buchan RJ Cook SA. MicroRNA-223 regulates Glut4 expression and cardiomyocyte glucose metabolism. Cardiovasc Res. (2010) 86:410–20. 10.1093/cvr/cvq010 20080987

[27] Pavlova NN Thompson CB. The emerging hallmarks of cancer metabolism. Cell Metab. (2016) 23:27–47. 10.1016/j.cmet.2015.12.006 26771115 PMC4715268

[28] Oermann EK Wu J Guan K-L Xiong Y. Alterations of metabolic genes and metabolites in cancer. Semin Cell Dev Biol. (2012) 23:370–80. 10.1016/j.semcdb.2012.01.013 22306135 PMC3351496

[29] Chen D Wang H Chen J Li Z Li S Hu Z. MicroRNA-129-5p Regulates glycolysis and cell proliferation by targeting the glucose transporter SLC2A3 in gastric cancer cells. Front Pharmacol. (2018) 9:502. 10.3389/fphar.2018.00502 29867504 PMC5962750

[30] Guo W Qiu Z Wang Z Wang Q Tan N Chen T. MiR-199a-5p is negatively associated with malignancies and regulates glycolysis and lactate production by targeting hexokinase 2 in liver cancer. Hepatol Baltim Md. (2015) 62:1132–44. 10.1002/hep.27929 26054020

[31] Yang Y Ishak Gabra MB Hanse EA Lowman XH Tran TQ Li H. MiR-135 suppresses glycolysis and promotes pancreatic cancer cell adaptation to metabolic stress by targeting phosphofructokinase-1. Nat Commun. (2019) 10:809. 10.1038/s41467-019-08759-0 30778058 PMC6379428

[32] Zhang X Li Z Xuan Z Xu P Wang W Chen Z. Novel role of miR-133a-3p in repressing gastric cancer growth and metastasis via blocking autophagy-mediated glutaminolysis. J Exp Clin Cancer Res CR. (2018) 37:320. 10.1186/s13046-018-0993-y 30572959 PMC6302516

[33] Esau C Davis S Murray SF Yu XX Pandey SK Pear M. miR-122 regulation of lipid metabolism revealed by *in vivo* antisense targeting. Cell Metab. (2006) 3:87–98. 10.1016/j.cmet.2006.01.005 16459310

[34] DeBerardinis RJ Chandel NS. Fundamentals of cancer metabolism. Sci Adv. (2016) 2:e1600200. 10.1126/sciadv.1600200 27386546 PMC4928883

[35] Dang CV. Links between metabolism and cancer. Genes Dev. (2012) 26:877–90. 10.1101/gad.189365.112 22549953 PMC3347786

[36] Levine AJ Puzio-Kuter AM. The control of the metabolic switch in cancers by oncogenes and tumor suppressor genes. Science. (2010) 330:1340–4. 10.1126/science.1193494 21127244

[37] Vaupel P Schmidberger H Mayer A. The Warburg effect: essential part of metabolic reprogramming and central contributor to cancer progression. Int J Radiat Biol. (2019) 95:912–9. 10.1080/09553002.2019.1589653 30822194

[38] Vander Heiden MG Cantley LC Thompson CB. Understanding the Warburg effect: the metabolic requirements of cell proliferation. Science. (2009) 324:1029–33. 10.1126/science.1160809 19460998 PMC2849637

[39] Lunt SY Vander Heiden MG. Aerobic glycolysis: meeting the metabolic requirements of cell proliferation. Annu Rev Cell Dev Biol. (2011) 27:441–64. 10.1146/annurev-cellbio-092910-154237 21985671

[40] Lebelo MT Joubert AM Visagie MH. Warburg effect and its role in tumourigenesis. Arch Pharm Res. (2019) 42:833–47. 10.1007/s12272-019-01185-2 31473944

[41] Cairns RA Harris IS Mak TW. Regulation of cancer cell metabolism. Nat Rev Cancer. (2011) 11:85–95. 10.1038/nrc2981 21258394

[42] Wu W Zhao S. Metabolic changes in cancer: beyond the Warburg effect. Acta Biochim Biophys Sin. (2013) 45:18–26. 10.1093/abbs/gms104 23257292

[43] Soga T. Cancer metabolism: key players in metabolic reprogramming. Cancer Sci. (2013) 104:275–81. 10.1111/cas.12085 23279446 PMC7657261

[44] Kato Y Maeda T Suzuki A Baba Y. Cancer metabolism: new insights into classic characteristics. Jpn Dent Sci Rev. (2018) 54:8–21. 10.1016/j.jdsr.2017.08.003 29628997 PMC5884251

[45] Li Z Zhang H. Reprogramming of glucose, fatty acid and amino acid metabolism for cancer progression. Cell Mol Life Sci CMLS. (2016) 73:377–92. 10.1007/s00018-015-2070-4 26499846 PMC11108301

[46] Li C Zhang G Zhao L Ma Z Chen H. Metabolic reprogramming in cancer cells: glycolysis, glutaminolysis, and Bcl-2 proteins as novel therapeutic targets for cancer. World J Surg Oncol. (2016) 14:15. 10.1186/s12957-016-0769-9 26791262 PMC4721116

[47] Schwörer S Vardhana SA Thompson CB. Cancer metabolism drives a stromal regenerative response. Cell Metab. (2019) 29:576–91. 10.1016/j.cmet.2019.01.015 30773467 PMC6692899

[48] Ippolito L Morandi A Taddei ML Parri M Comito G Iscaro A. Cancer-associated fibroblasts promote prostate cancer malignancy via metabolic rewiring and mitochondrial transfer. Oncogene. (2019) 38:5339–55. 10.1038/s41388-019-0805-7 30936458

[49] Netea-Maier RT Smit JWA Netea MG. Metabolic changes in tumor cells and tumor-associated macrophages: a mutual relationship. Cancer Lett. (2018) 413:102–9. 10.1016/j.canlet.2017.10.037 29111350

[50] Papa S Choy PM Bubici C. The ERK and JNK pathways in the regulation of metabolic reprogramming. Oncogene. (2019) 38:2223–40. 10.1038/s41388-018-0582-8 30487597 PMC6398583

[51] Yeung SJ Pan J Lee M-H. Roles of p53, MYC and HIF-1 in regulating glycolysis - the seventh hallmark of cancer. Cell Mol Life Sci CMLS. (2008) 65:3981–99. 10.1007/s00018-008-8224-x 18766298 PMC11131737

[52] Zwaans BMM Lombard DB. Interplay between sirtuins, MYC and hypoxia-inducible factor in cancer-associated metabolic reprogramming. Dis Model Mech. (2014) 7:1023–32. 10.1242/dmm.016287 25085992 PMC4142723

[53] Mueckler M Thorens B. The SLC2 (GLUT) family of membrane transporters. Mol Aspects Med. (2013) 34:121–38. 10.1016/j.mam.2012.07.001 23506862 PMC4104978

[54] Thorens B Mueckler M. Glucose transporters in the 21st century. Am J Physiol Endocrinol Metab. (2010) 298:E141–5. 10.1152/ajpendo.00712.2009 20009031 PMC2822486

[55] Liu M Gao J Huang Q Jin Y Wei Z. Downregulating microRNA-144 mediates a metabolic shift in lung cancer cells by regulating GLUT1 expression. Oncol Lett. (2016) 11:3772–6. 10.3892/ol.2016.4468 27313692 PMC4888127

[56] Qu W Ding S-M Cao G Wang S-J Zheng X-H Li G-H. miR-132 mediates a metabolic shift in prostate cancer cells by targeting Glut1. FEBS Open Bio. (2016) 6:735–41. 10.1002/2211-5463.12086 27398313 PMC4932453

[57] King BC Esguerra JLS Golec E Eliasson L Kemper C Blom AM. CD46 activation regulates miR-150–mediated control of GLUT1 expression and cytokine secretion in human CD4 ^+^ T Cells. J Immunol. (2016) 196:1636–45. 10.4049/jimmunol.1500516 26746193 PMC4745132

[58] Chow T-FF Mankaruos M Scorilas A Youssef Y Girgis A Mossad S. The miR-17-92 cluster is over expressed in and has an oncogenic effect on renal cell carcinoma. J Urol. (2010) 183:743–51. 10.1016/j.juro.2009.09.086 20022054

[59] Fei X Qi M Wu B Song Y Wang Y Li T. MicroRNA-195-5p suppresses glucose uptake and proliferation of human bladder cancer T24 cells by regulating GLUT3 expression. FEBS Lett. (2012) 586:392–7. 10.1016/j.febslet.2012.01.006 22265971

[60] Dai D-W Lu Q Wang L-X Zhao W-Y Cao Y-Q Li Y-N. Decreased miR-106a inhibits glioma cell glucose uptake and proliferation by targeting SLC2A3 in GBM. BMC Cancer. (2013) 13:478. 10.1186/1471-2407-13-478 24124917 PMC3853007

[61] Horie T Ono K Nishi H Iwanaga Y Nagao K Kinoshita M. MicroRNA-133 regulates the expression of GLUT4 by targeting KLF15 and is involved in metabolic control in cardiac myocytes. Biochem Biophys Res Commun. (2009) 389:315–20. 10.1016/j.bbrc.2009.08.136 19720047

[62] Ling H-Y Hu B Hu X-B Zhong J Feng S-D Qin L. MiRNA-21 reverses high glucose and high insulin induced insulin resistance in 3T3-L1 adipocytes through targeting phosphatase and tensin homologue. Exp Clin Endocrinol Diabetes Off J Ger Soc Endocrinol Ger Diabetes Assoc. (2012) 120:553–9. 10.1055/s-0032-1311644 22956257

[63] Raychaudhuri S. MicroRNAs overexpressed in growth-restricted rat skeletal muscles regulate the glucose transport in cell culture targeting central TGF-β factor SMAD4. PLoS ONE. (2012) 7:e34596. 10.1371/journal.pone.0034596 22506032 PMC3323545

[64] Esteves JV Enguita FJ Machado UF. MicroRNAs-mediated regulation of skeletal muscle GLUT4 expression and translocation in insulin resistance. J Diabetes Res. (2017) 2017:7267910. 10.1155/2017/7267910 28428964 PMC5385897

[65] Yin C Qie S Sang N. Carbon source metabolism and its regulation in cancer cells. Crit Rev Eukaryot Gene Expr. (2012) 22:17–35. 10.1615/critreveukargeneexpr.v22.i1.20 22339657 PMC4505802

[66] Dang CV Glutaminolysis: supplying carbon or nitrogen or both for cancer cells? Cell Cycle Georget Tex. (2010) 9:3884–6. 10.4161/cc.9.19.13302 20948290

[67] John S Weiss JN Ribalet B. Subcellular localization of hexokinases I and II directs the metabolic fate of glucose. PLoS ONE. (2011) 6:e17674. 10.1371/journal.pone.0017674 21408025 PMC3052386

[68] Mandarino LJ Printz RL Cusi KA Kinchington P O'Doherty RM Osawa H. Regulation of hexokinase II and glycogen synthase mRNA, protein, and activity in human muscle. Am J Physiol. (1995) 269:E701–8. 10.1152/ajpendo.1995.269.4.E701 7485484

[69] Wyatt E Wu R Rabeh W Park H-W Ghanefar M Ardehali H. Regulation and cytoprotective role of hexokinase III. PLoS ONE. (2010) 5:e13823. 10.1371/journal.pone.0013823 21072205 PMC2972215

[70] Yasuda S Arii S Mori A Isobe N Yang W Oe H Hexokinase II and VEGF expression in liver tumors: correlation with hypoxia-inducible factor-1α and its significance. J Hepatol. (2004) 40:117–23. 10.1016/S0168-8278(03)00503-8 14672622

[71] Jiao L Zhang H-L Li D-D Yang K-L Tang J Li X. Regulation of glycolytic metabolism by autophagy in liver cancer involves selective autophagic degradation of HK2 (hexokinase 2). Autophagy. (2018) 14:671–84. 10.1080/15548627.2017.1381804 28980855 PMC5959330

[72] Palmieri D Fitzgerald D Shreeve SM Hua E Bronder JL Weil RJ. Analyses of resected human brain metastases of breast cancer reveal the association between up-regulation of hexokinase 2 and poor prognosis. Mol Cancer Res. (2009) 7:1438–45. 10.1158/1541-7786.MCR-09-0234 19723875 PMC2746883

[73] Fang R Xiao T Fang Z Sun Y Li F Gao Y. MicroRNA-143 (miR-143) regulates cancer glycolysis via targeting hexokinase 2 gene. J Biol Chem. (2012) 287:23227–35. 10.1074/jbc.M112.373084 22593586 PMC3391126

[74] Peschiaroli A Giacobbe A Formosa A Markert EK Bongiorno-Borbone L Levine AJ. miR-143 regulates hexokinase 2 expression in cancer cells. Oncogene. (2013) 32:797–802. 10.1038/onc.2012.100 22469988

[75] Gregersen LH Jacobsen A Frankel LB Wen J Krogh A Lund AH. MicroRNA-143 down-regulates Hexokinase 2 in colon cancer cells. BMC Cancer. (2012) 12:232. 10.1186/1471-2407-12-232 22691140 PMC3480834

[76] Li L-Q Yang Y Chen H Zhang L Pan D Xie W-J. MicroRNA-181b inhibits glycolysis in gastric cancer cells via targeting hexokinase 2 gene. Cancer Biomark Sect Dis Markers. (2016) 17:75–81. 10.3233/CBM-160619 27314295 PMC13020476

[77] Jiang S Zhang L-F Zhang H-W Hu S Lu M-H Liang S. A novel miR-155/miR-143 cascade controls glycolysis by regulating hexokinase 2 in breast cancer cells. EMBO J. (2012) 31:1985–98. 10.1038/emboj.2012.45 22354042 PMC3343331

[78] Ahmad A Aboukameel A Kong D Wang Z Sethi S Chen W. Phosphoglucose isomerase/autocrine motility factor mediates epithelial-mesenchymal transition regulated by miR-200 in breast cancer cells. Cancer Res. (2011) 71:3400–9. 10.1158/0008-5472.CAN-10-0965 21389093 PMC3085607

[79] Rengaraj D Park TS Lee SI Lee BR Han BK Song G. Regulation of glucose phosphate isomerase by the 3'UTR-specific miRNAs miR-302b and miR-17-5p in chicken primordial germ cells. Biol Reprod. (2013) 89:33. 10.1095/biolreprod.112.105692 23825378

[80] Fabani MM Gait MJ. miR-122 targeting with LNA/2'-O-methyl oligonucleotide mixmers, peptide nucleic acids (PNA), and PNA-peptide conjugates. RNA NYN. (2008) 14:336–46. 10.1261/rna.844108 18073344 PMC2212241

[81] Calin GA Cimmino A Fabbri M Ferracin M Wojcik SE Shimizu M. MiR-15a and miR-16-1 cluster functions in human leukemia. Proc Natl Acad Sci USA. (2008) 105:5166–71. 10.1073/pnas.0800121105 18362358 PMC2278188

[82] Montoya V Fan H Bryar PJ Weinstein JL Mets MB Feng G. Novel miRNA-31 and miRNA-200a-mediated regulation of retinoblastoma proliferation. PLoS ONE. (2015) 10:e0138366. 10.1371/journal.pone.0138366 26379276 PMC4574557

[83] Sikand K Singh J Ebron JS Shukla GC. Housekeeping gene selection advisory: glyceraldehyde-3-phosphate dehydrogenase (GAPDH) and β-actin are targets of miR-644a. PLoS ONE. (2012) 7:e47510. 10.1371/journal.pone.0047510 23091630 PMC3472982

[84] Khanna M Saini S Shariff M Ronsard L Singh JK Kumar H. Data highlighting miR-155 and GAPDH correlation. Data Brief. (2019) 24:103945. 10.1016/j.dib.2019.103945 31193288 PMC6523032

[85] Wong T-S Liu X-B Chung-Wai Ho A Po-Wing Yuen A Wai-Man Ng R Ignace Wei W. Identification of pyruvate kinase type M2 as potential oncoprotein in squamous cell carcinoma of tongue through microRNA profiling. Int J Cancer. (2008) 123:251–7. 10.1002/ijc.23583 18464261

[86] Kefas B Comeau L Erdle N Montgomery E Amos S Purow B. Pyruvate kinase M2 is a target of the tumor-suppressive microRNA-326 and regulates the survival of glioma cells. Neuro-Oncol. (2010) 12:1102–12. 10.1093/neuonc/noq080 20667897 PMC3098027

[87] Liu AM Xu Z Shek FH Wong K-F Lee NP Poon RT. miR-122 targets pyruvate kinase M2 and affects metabolism of hepatocellular carcinoma. PLoS ONE. (2014) 9:e86872. 10.1371/journal.pone.0086872 24466275 PMC3900676

[88] Sun Y Zhao X Zhou Y Hu Y. miR-124, miR-137 and miR-340 regulate colorectal cancer growth via inhibition of the Warburg effect. Oncol Rep. (2012) 28:1346–52. 10.3892/or.2012.1958 22895557

[89] Li W Wang J Chen Q-D Qian X Li Q Yin Y. Insulin promotes glucose consumption via regulation of miR-99a/mTOR/PKM2 pathway. PLoS ONE. (2013) 8:e64924. 10.1371/journal.pone.0064924 23762265 PMC3677911

[90] Feng Y Xiong Y Qiao T Li X Jia L Han Y. Lactate dehydrogenase A: a key player in carcinogenesis and potential target in cancer therapy. Cancer Med. (2018) 7:6124–36. 10.1002/cam4.1820 30403008 PMC6308051

[91] Li J Zhu S Tong J Hao H Yang J Liu Z. Suppression of lactate dehydrogenase A compromises tumor progression by downregulation of the Warburg effect in glioblastoma. Neuroreport. (2016) 27:110–5. 10.1097/WNR.0000000000000506 26694942 PMC4712768

[92] Dong T Liu Z Xuan Q Wang Z Ma W Zhang Q. Tumor LDH-A expression and serum LDH status are two metabolic predictors for triple negative breast cancer brain metastasis. Sci Rep. (2017) 7:6069. 10.1038/s41598-017-06378-7 28729678 PMC5519725

[93] Kinoshita T Nohata N Yoshino H Hanazawa T Kikkawa N Fujimura L. Tumor suppressive microRNA-375 regulates lactate dehydrogenase B in maxillary sinus squamous cell carcinoma. Int J Oncol. (2012) 40:185–93. 10.3892/ijo.2011.1196 21922130

[94] Xiao X Huang X Ye F Chen B Song C Wen J. The miR-34a-LDHA axis regulates glucose metabolism and tumor growth in breast cancer. Sci Rep. (2016) 6:21735. 10.1038/srep21735 26902416 PMC4763192

[95] Chen H Gao S Cheng C. MiR-323a-3p suppressed the glycolysis of osteosarcoma via targeting LDHA. Hum Cell. (2018) 31:300–9. 10.1007/s13577-018-0215-0 30088225

[96] Li L Kang L Zhao W Feng Y Liu W Wang T. miR-30a-5p suppresses breast tumor growth and metastasis through inhibition of LDHA-mediated Warburg effect. Cancer Lett. (2017) 400:89–98. 10.1016/j.canlet.2017.04.034 28461244

[97] Wang J Wang H Liu A Fang C Hao J Wang Z. Lactate dehydrogenase A negatively regulated by miRNAs promotes aerobic glycolysis and is increased in colorectal cancer. Oncotarget. (2015) 6:19456–468. 10.18632/oncotarget.3318 26062441 PMC4637298

[98] Yuan D Zheng S Wang L Li J Yang J Wang B. MiR-200c inhibits bladder cancer progression by targeting lactate dehydrogenase A. Oncotarget. (2017) 8:67663–9. 10.18632/oncotarget.18801 28978061 PMC5620201

[99] He Y Chen X Yu Y Li J Hu Q Xue C. LDHA is a direct target of miR-30d-5p and contributes to aggressive progression of gallbladder carcinoma. Mol Carcinog. (2018) 57:772–83. 10.1002/mc.22799 29569755

[100] Halestrap AP. The SLC16 gene family - structure, role and regulation in health and disease. Mol Aspects Med. (2013) 34:337–49. 10.1016/j.mam.2012.05.003 23506875

[101] Pullen TJ da Silva Xavier G Kelsey G Rutter GA. miR-29a and miR-29b contribute to pancreatic beta-cell-specific silencing of monocarboxylate transporter 1 (Mct1). Mol Cell Biol. (2011) 31:3182–94. 10.1128/MCB.01433-10 21646425 PMC3147603

[102] Liang D Zhang Y Han J Wang W Liu Y Li J. Embryonic stem cell-derived pancreatic endoderm transplant with MCT1-suppressing miR-495 attenuates type II diabetes in mice. Endocr J. (2015) 62:907–20. 10.1507/endocrj.EJ15-0186 26211669

[103] Romero-Cordoba SL Rodriguez-Cuevas S Bautista-Pina V Maffuz-Aziz A D'Ippolito E Cosentino G. Loss of function of miR-342-3p results in MCT1 over-expression and contributes to oncogenic metabolic reprogramming in triple negative breast cancer. Sci Rep. (2018) 8:12252. 10.1038/s41598-018-29708-9 30115973 PMC6095912

[104] Zhao Y Li W Li M Hu Y Zhang H Song G. Targeted inhibition of MCT4 disrupts intracellular pH homeostasis and confers self-regulated apoptosis on hepatocellular carcinoma. Exp Cell Res. (2019)111591. 10.1016/j.yexcr.2019.111591 31479685

[105] Hensley CT Wasti AT DeBerardinis RJ. Glutamine and cancer: cell biology, physiology, and clinical opportunities. J Clin Invest. (2013) 123:3678–84. 10.1172/JCI69600 23999442 PMC3754270

[106] Daye D Wellen KE. Metabolic reprogramming in cancer: unraveling the role of glutamine in tumorigenesis. Semin Cell Dev Biol. (2012) 23:362–9. 10.1016/j.semcdb.2012.02.002 22349059

[107] Dong J Xiao D Zhao Z Ren P Li C Hu Y. Epigenetic silencing of microRNA-137 enhances ASCT2 expression and tumor glutamine metabolism. Oncogenesis. (2017) 6:e356. 10.1038/oncsis.2017.59 28692032 PMC5541711

[108] Gao P Tchernyshyov I Chang T-C Lee Y-S Kita K Ochi T. c-Myc suppression of miR-23a/b enhances mitochondrial glutaminase expression and glutamine metabolism. Nature. (2009) 458:762–5. 10.1038/nature07823 19219026 PMC2729443

[109] Chang X Zhu W Zhang H Lian S. Sensitization of melanoma cells to temozolomide by overexpression of microRNA 203 through direct targeting of glutaminase-mediated glutamine metabolism. Clin Exp Dermatol. (2017) 42:614–21. 10.1111/ced.13119 28597996

[110] Anderton B Camarda R Balakrishnan S Balakrishnan A Kohnz RA Lim L. MYC-driven inhibition of the glutamate-cysteine ligase promotes glutathione depletion in liver cancer. EMBO Rep. (2017) 18:569–85. 10.15252/embr.201643068 28219903 PMC5376764

[111] Liu Z Wang J Li Y Fan J Chen L Xu R. MicroRNA-153 regulates glutamine metabolism in glioblastoma through targeting glutaminase. Tumour Biol J Int Soc Oncodevelopmental Biol Med. (2017) 39:1010428317691429. 10.1177/1010428317691429 28218035

[112] Muys BR Sousa JF Plaça JR de Araújo LF Sarshad AA Anastasakis DG. miR-450a acts as a tumor suppressor in ovarian cancer by regulating energy metabolism. Cancer Res. (2019) 79:3294–305. 10.1158/0008-5472.CAN-19-0490 31101765 PMC6606360

[113] Zacksenhaus E Shrestha M Liu JC Vorobieva I Chung PED Ju Y. Mitochondrial OXPHOS induced by RB1 deficiency in breast cancer: implications for anabolic metabolism, stemness, and metastasis. Trends Cancer. (2017) 3:768–79. 10.1016/j.trecan.2017.09.002 29120753

[114] Marchiq I Le Floch R Roux D Simon M-P Pouyssegur J. Genetic disruption of lactate/H+ symporters (MCTs) and their subunit CD147/BASIGIN sensitizes glycolytic tumor cells to phenformin. Cancer Res. (2015) 75:171–80. 10.1158/0008-5472.CAN-14-2260 25403912

[115] Ippolito L Marini A Cavallini L Morandi A Pietrovito L Pintus G. Metabolic shift toward oxidative phosphorylation in docetaxel resistant prostate cancer cells. Oncotarget. (2016) 7:61890–904. 10.18632/oncotarget.11301 27542265 PMC5308698

[116] Patel B Gove CD Hutchinson WL Mowbray J. Substantial quantities of the high energy derivative oligophosphoglyceroyl-ATP are located in mitochondria in rat liver. Biochim Biophys Acta. (1991) 1074:178–81. 10.1016/0304-4165(91)90058-o 2043668

[117] Muralimanoharan S Maloyan A Mele J Guo C Myatt LG Myatt L. MIR-210 modulates mitochondrial respiration in placenta with preeclampsia. Placenta. (2012) 33:816–23. 10.1016/j.placenta.2012.07.002 22840297 PMC3439551

[118] Das S Ferlito M Kent OA Fox-Talbot K Wang R Liu D. Nuclear miRNA regulates the mitochondrial genome in the heart. Circ Res. (2012) 110:1596–603. 10.1161/CIRCRESAHA.112.267732 22518031 PMC3390752

[119] Aschrafi A Schwechter AD Mameza MG Natera-Naranjo O Gioio AE Kaplan BB. MicroRNA-338 regulates local cytochrome c oxidase IV mRNA levels and oxidative phosphorylation in the axons of sympathetic neurons. J Neurosci. (2008) 28:12581–90. 10.1523/JNEUROSCI.3338-08.2008 19020050 PMC3496265

[120] Willers IM Martínez-Reyes I Martínez-Diez M Cuezva JM. miR-127-5p targets the 3'UTR of human β-F1-ATPase mRNA and inhibits its translation. Biochim Biophys Acta. (2012) 1817:838–48. 10.1016/j.bbabio.2012.03.005 22433606

[121] Baseler WA Thapa D Jagannathan R Dabkowski ER Croston TL Hollander JM. miR-141 as a regulator of the mitochondrial phosphate carrier (Slc25a3) in the type 1 diabetic heart. Am J Physiol Cell Physiol. (2012) 303:C1244–51. 10.1152/ajpcell.00137.2012 23034391 PMC3532490

[122] Pucci S Mazzarelli P. MicroRNA Dysregulation in colon cancer microenvironment interactions: the importance of small things in metastases. Cancer Microenviron. (2011) 4:155–62. 10.1007/s12307-011-0062-y 21909877 PMC3170419

[123] Morton MJ Pitel ML. Reflex sympathetic dystrophy syndrome complicating the management of TMJ symptoms. A case report. Cranio J Craniomandib Pract. (1989) 7:239–42. 2638214 10.1080/08869634.1989.11678291

[124] Wise DR DeBerardinis RJ Mancuso A Sayed N Zhang X-Y Pfeiffer HK. Myc regulates a transcriptional program that stimulates mitochondrial glutaminolysis and leads to glutamine addiction. Proc Natl Acad Sci USA. (2008) 105:18782–7. 10.1073/pnas.0810199105 19033189 PMC2596212

[125] Kimmelman AC. Metabolic dependencies in RAS-driven cancers. Clin Cancer Res Off J Am Assoc Cancer Res. (2015) 21:1828–34. 10.1158/1078-0432.CCR-14-2425 25878364 PMC4400826

[126] Simabuco FM Morale MG Pavan ICB Morelli AP Silva FR Tamura RE p53 and metabolism: from mechanism to therapeutics. Oncotarget. (2018) 9:23780–823. 10.18632/oncotarget.25267 29805774 PMC5955117

[127] Jones RG Thompson CB. Tumor suppressors and cell metabolism: a recipe for cancer growth. Genes Dev. (2009) 23:537–48. 10.1101/gad.1756509 19270154 PMC2763495

[128] Catanzaro G Besharat ZM Miele E Chiacchiarini M Po A Carai A. The miR-139-5p regulates proliferation of supratentorial paediatric low-grade gliomas by targeting the PI3K/AKT/mTORC1 signalling. Neuropathol Appl Neurobiol. (2018) 44:687–706. 10.1111/nan.12479 29478280

[129] Koh H-J Toyoda T Fujii N Jung MM Rathod A Middelbeek RJ-W. Sucrose nonfermenting AMPK-related kinase (SNARK) mediates contraction-stimulated glucose transport in mouse skeletal muscle. Proc Natl Acad Sci USA. (2010) 107:15541–6. 10.1073/pnas.1008131107 20713714 PMC2932588

[130] Dávalos A Goedeke L Smibert P Ramírez CM Warrier NP Andreo U. miR-33a/b contribute to the regulation of fatty acid metabolism and insulin signaling. Proc Natl Acad Sci USA. (2011) 108:9232–7. 10.1073/pnas.1102281108 21576456 PMC3107310

[131] Mersey BD Jin P Danner DJ. Human microRNA (miR29b) expression controls the amount of branched chain alpha-ketoacid dehydrogenase complex in a cell. Hum Mol Genet. (2005) 14:3371–7. 10.1093/hmg/ddi368 16203741

[132] Ward PS Thompson CB Metabolic reprogramming: a cancer hallmark even Warburg did not anticipate. Cancer Cell. (2012) 21:297–308. 10.1016/j.ccr.2012.02.014 22439925 PMC3311998

[133] Chen X Yang F Zhang T Wang W Xi W Li Y. MiR-9 promotes tumorigenesis and angiogenesis and is activated by MYC and OCT4 in human glioma. J Exp Clin Cancer Res CR. (2019) 38:99. 10.1186/s13046-019-1078-2 30795814 PMC6385476

[134] Tomasetti M Lee W Santarelli L Neuzil J. Exosome-derived microRNAs in cancer metabolism: possible implications in cancer diagnostics and therapy. Exp Mol Med. (2017) 49:e285. 10.1038/emm.2016.153 28104913 PMC5291842

[135] Tomasetti M Santarelli L Neuzil J Dong L. MicroRNA regulation of cancer metabolism: role in tumour suppression. Mitochondrion. (2014) 19(Pt A):29–38. 10.1016/j.mito.2014.06.004 24960472

[136] Shen G Li X Jia Y Piazza GA Xi Y. Hypoxia-regulated microRNAs in human cancer. Acta Pharmacol Sin. (2013) 34:336–41. 10.1038/aps.2012.195 23377548 PMC3587916

[137] Al Tameemi W Dale TP Al-Jumaily RMK Forsyth NR. Hypoxia-modified cancer cell metabolism. Front Cell Dev Biol. (2019) 7:4. 10.3389/fcell.2019.00004 30761299 PMC6362613

[138] English SG Hadj-Moussa H Storey KB. MicroRNAs regulate survival in oxygen-deprived environments. J Exp Biol. (2018) 221(Pt 23):jeb190579. 10.1242/jeb.190579 30322977

[139] Papandreou I Cairns RA Fontana L Lim AL Denko NC. HIF-1 mediates adaptation to hypoxia by actively downregulating mitochondrial oxygen consumption. Cell Metab. (2006) 3:187–97. 10.1016/j.cmet.2006.01.012 16517406

[140] Kim J Tchernyshyov I Semenza GL Dang CV. HIF-1-mediated expression of pyruvate dehydrogenase kinase: a metabolic switch required for cellular adaptation to hypoxia. Cell Metab. (2006) 3:177–85. 10.1016/j.cmet.2006.02.002 16517405

[141] Hagen T. Oxygen versus reactive oxygen in the regulation of HIF-1α: the balance tips. Biochem Res Int. (2012) 2012:436981. 10.1155/2012/436981 23091723 PMC3474226

[142] Marín-Hernández A Gallardo-Pérez JC Ralph SJ Rodríguez-Enríquez S Moreno-Sánchez R. HIF-1alpha modulates energy metabolism in cancer cells by inducing over-expression of specific glycolytic isoforms. Mini Rev Med Chem. (2009) 9:1084–101. 10.2174/138955709788922610 19689405

[143] Nagao A Kobayashi M Koyasu S Chow CCT Harada H. HIF-1-Dependent reprogramming of glucose metabolic pathway of cancer cells and its therapeutic significance. Int J Mol Sci. (2019) 20:238. 10.3390/ijms20020238 30634433 PMC6359724

[144] Singh D Arora R Kaur P Singh B Mannan R Arora S. Overexpression of hypoxia-inducible factor and metabolic pathways: possible targets of cancer. Cell Biosci. (2017) 7:62. 10.1186/s13578-017-0190-2 29158891 PMC5683220

[145] Tokuda K Baron B Yamashiro C Kuramitsu Y Kitagawa T Kobayashi M. Up-regulation of the pentose phosphate pathway and HIF-1α expression during neural progenitor cell induction following glutamate treatment in rat *ex vivo* retina. Cell Biol Int. (2019) 10.1002/cbin.11212. [Epub ahead of print]. 31393075

[146] Gordan JD Thompson CB Simon MC. HIF and c-Myc: sibling rivals for control of cancer cell metabolism and proliferation. Cancer Cell. (2007) 12:108–13. 10.1016/j.ccr.2007.07.006 17692803 PMC3215289

[147] Podar K Anderson KC. A therapeutic role for targeting c-Myc/Hif-1-dependent signaling pathways. Cell Cycle Georget Tex. (2010) 9:1722–8. 10.4161/cc.9.9.11358 20404562 PMC3155944

[148] Zhang H Gao P Fukuda R Kumar G Krishnamachary B Zeller KI. HIF-1 inhibits mitochondrial biogenesis and cellular respiration in VHL-deficient renal cell carcinoma by repression of C-MYC activity. Cancer Cell. (2007) 11:407–20. 10.1016/j.ccr.2007.04.001 17482131

[149] Kim J Gao P Liu Y-C Semenza GL Dang CV. Hypoxia-inducible factor 1 and dysregulated c-Myc cooperatively induce vascular endothelial growth factor and metabolic switches hexokinase 2 and pyruvate dehydrogenase kinase 1. Mol Cell Biol. (2007) 27:7381–93. 10.1128/MCB.00440-07 17785433 PMC2169056

[150] Amelio I Mancini M Petrova V Cairns RA Vikhreva P Nicolai S. p53 mutants cooperate with HIF-1 in transcriptional regulation of extracellular matrix components to promote tumor progression. Proc Natl Acad Sci USA. (2018) 115:E10869–78. 10.1073/pnas.1808314115 30381462 PMC6243248

[151] Obacz J Pastorekova S Vojtesek B Hrstka R. Cross-talk between HIF and p53 as mediators of molecular responses to physiological and genotoxic stresses. Mol Cancer. (2013) 12:93. 10.1186/1476-4598-12-93 23945296 PMC3844392

[152] Jin F Wang Y Zhu Y Li S Liu Y Chen C. The miR-125a/HK2 axis regulates cancer cell energy metabolism reprogramming in hepatocellular carcinoma. Sci Rep. (2017) 7:3089. 10.1038/s41598-017-03407-3 28596599 PMC5465066

[153] Lin YX An FM Zhan Q. [Research progress of microRNAs in regulation of lipid metabolism-related proteins for influencing the pathogenesis of hepatocellular carcinoma]. Zhonghua Gan Zang Bing Za Zhi Zhonghua Ganzangbing Zazhi Chin J Hepatol. (2019) 27:219–22. 10.3760/cma.j.issn.1007-3418.2019.03.011 30929341 PMC12814129

[154] Nie Z-Y Liu X-J Zhan Y Liu M-H Zhang X-Y Li Z-Y. miR-140-5p induces cell apoptosis and decreases Warburg effect in chronic myeloid leukemia by targeting SIX1. Biosci Rep. (2019) 39:BSR20190150. 10.1042/BSR20190150 30962263 PMC6488949

[155] Krokker L Nyíro G Reiniger L Darvasi O Szücs N Czirják S. Differentially expressed miRNAs influence metabolic processes in pituitary oncocytoma. Neurochem Res. (2019) 44:2360–71. 10.1007/s11064-019-02789-2 30945144 PMC6776564

[156] Xu T Zhang K Shi J Huang B Wang X Qian K. MicroRNA-940 inhibits glioma progression by blocking mitochondrial folate metabolism through targeting of MTHFD2. Am J Cancer Res. (2019) 9:250–69. 30906627 PMC6405966

[157] De Santi C Melaiu O Bonotti A Cascione L Di Leva G Foddis R. Deregulation of miRNAs in malignant pleural mesothelioma is associated with prognosis and suggests an alteration of cell metabolism. Sci Rep. (2017) 7:3140. 10.1038/s41598-017-02694-0 28600498 PMC5466648

[158] Ling Z Liu D Zhang G Liang Q Xiang P Xu Y. miR-361-5p modulates metabolism and autophagy via the Sp1-mediated regulation of PKM2 in prostate cancer. Oncol Rep. (2017) 38:1621–8. 10.3892/or.2017.5852 29094170

[159] Alfardus H McIntyre A Smith S. MicroRNA regulation of glycolytic metabolism in glioblastoma. BioMed Res Int. (2017) 2017:9157370. 10.1155/2017/9157370 28804724 PMC5539934

[160] Guo H Nan Y Zhen Y Zhang Y Guo L Yu K. miRNA-451 inhibits glioma cell proliferation and invasion by downregulating glucose transporter 1. Tumour Biol. (2016) 37:13751–61. 10.1007/s13277-016-5219-3 27476171

[161] Fong MY Zhou W Liu L Alontaga AY Chandra M Ashby J. Breast-cancer-secreted miR-122 reprograms glucose metabolism in premetastatic niche to promote metastasis. Nat Cell Biol. (2015) 17:183–94. 10.1038/ncb3094 25621950 PMC4380143

[162] Eichner LJ Perry M-C Dufour CR Bertos N Park M St-Pierre J. miR-378(*) mediates metabolic shift in breast cancer cells via the PGC-1β/ERRγ transcriptional pathway. Cell Metab. (2010) 12:352–61. 10.1016/j.cmet.2010.09.002 20889127

[163] Hatziapostolou M Polytarchou C Iliopoulos D. miRNAs link metabolic reprogramming to oncogenesis. Trends Endocrinol Metab. (2013) 24:361–73. 10.1016/j.tem.2013.03.002 23602813

[164] Vernieri C Casola S Foiani M Pietrantonio F de Braud F Longo V. Targeting cancer metabolism: dietary and pharmacologic interventions. Cancer Discov. (2016) 6:1315–33. 10.1158/2159-8290.CD-16-0615 27872127 PMC5140697

[165] Drakaki A Hatziapostolou M Iliopoulos D. Therapeutically targeting microRNAs in liver cancer. Curr Pharm Des. (2013) 19:1180–91. 10.2174/138161213804805658 23092338

[166] Nosengo N. Can you teach old drugs new tricks? Nature. (2016) 534:314–6. 10.1038/534314a 27306171

[167] Pantziarka P Bouche G Meheus L Sukhatme V Sukhatme VP Vikas P. The repurposing drugs in oncology (ReDO) project. Ecancermedicalscience. (2014) 8:442. 10.3332/ecancer.2014.442 25075216 PMC4096030

[168] Naidu S Garofalo M. microRNAs: an emerging paradigm in lung cancer chemoresistance. Front Med. (2015) 2:77. 10.3389/fmed.2015.00077 26583081 PMC4631988

[169] Ayers D Vandesompele J. Influence of microRNAs and long non-coding RNAs in cancer chemoresistance. Genes. (2017) 8:95. 10.3390/genes8030095 28273813 PMC5368699

[170] Oliveras-Ferraros C Cufí S Vazquez-Martin A Torres-Garcia VZ Del Barco S Martin-Castillo B. Micro(mi)RNA expression profile of breast cancer epithelial cells treated with the anti-diabetic drug metformin: induction of the tumor suppressor miRNA let-7a and suppression of the TGFβ-induced oncomiR miRNA-181a. Cell Cycle. (2011) 10:1144–51. 10.4161/cc.10.7.15210 21368581

[171] Bao B Wang Z Ali S Ahmad A Azmi AS Sarkar SH. Metformin inhibits cell proliferation, migration and invasion by attenuating CSC function mediated by deregulating miRNAs in pancreatic cancer cells. Cancer Prev Res. (2012) 5:355–64. 10.1158/1940-6207.CAPR-11-0299 22086681 PMC3786260

[172] Li W Yuan Y Huang L Qiao M Zhang Y. Metformin alters the expression profiles of microRNAs in human pancreatic cancer cells. Diabetes Res Clin Pract. (2012) 96:187–95. 10.1016/j.diabres.2011.12.028 22245693

[173] Wang F Xu J Liu H Liu Z Xia F. Metformin induces apoptosis by microRNA-26a-mediated downregulation of myeloid cell leukaemia-1 in human oral cancer cells. Mol Med Rep. (2016) 13:4671–6. 10.3892/mmr.2016.5143 27082123

[174] Yang F-Q Wang J-J Yan J-S Huang J-H Li W Che J-P. Metformin inhibits cell growth by upregulating microRNA-26a in renal cancer cells. Int J Clin Exp Med. (2014) 7:3289–96. 25419360 PMC4238495

[175] Cifarelli V Lashinger LM Devlin KL Dunlap SM Huang J Kaaks R. Metformin and rapamycin reduce pancreatic cancer growth in obese prediabetic mice by distinct microRNA-regulated mechanisms. Diabetes. (2015) 64:1632–42. 10.2337/db14-1132 25576058 PMC4407853

[176] Do MT Kim HG Choi JH Jeong HG. Metformin induces microRNA-34a to downregulate the Sirt1/Pgc-1α/Nrf2 pathway, leading to increased susceptibility of wild-type p53 cancer cells to oxidative stress and therapeutic agents. Free Radic Biol Med. (2014) 74:21–34. 10.1016/j.freeradbiomed.2014.06.010 24970682

[177] Xie W Wang L Sheng H Qiu J Zhang D Zhang L. Metformin induces growth inhibition and cell cycle arrest by upregulating microRNA34a in renal cancer cells. Med Sci Monit. (2017) 23:29–37. 10.12659/msm.898710 28045889 PMC5226302

[178] Wahdan-Alaswad RS Cochrane DR Spoelstra NS Howe EN Edgerton SM Anderson SM. Metformin-induced killing of triple-negative breast cancer cells is mediated by reduction in fatty acid synthase via miRNA-193b. Horm Cancer. (2014) 5:374–89. 10.1007/s12672-014-0188-8 25213330 PMC4570735

[179] Zhao W Zhang X Liu J Sun B Tang H Zhang H. miR-27a-mediated antiproliferative effects of metformin on the breast cancer cell line MCF-7. Oncol Rep. (2016) 36:3691–9. 10.3892/or.2016.5199 27779715

[180] Nangia-Makker P Yu Y Vasudevan A Farhana L Rajendra SG Levi E. Metformin: a potential therapeutic agent for recurrent colon cancer. PLoS ONE. (2014) 9:e84369. 10.1371/journal.pone.0084369 24465408 PMC3896365

[181] Cufí S Vazquez-Martin A Oliveras-Ferraros C Quirantes R Segura-Carretero A Micol V. Metformin lowers the threshold for stress-induced senescence: a role for the microRNA-200 family and miR-205. Cell Cycle. (2012) 11:1235–46. 10.4161/cc.11.6.19665 22356767

[182] Jiang X Ma N Wang D Li F He R Li D. Metformin inhibits tumor growth by regulating multiple miRNAs in human cholangiocarcinoma. Oncotarget. (2015) 6:3178–94. 10.18632/oncotarget.3063 25605008 PMC4413646

[183] Tseng H-W Li S-C Tsai K-W. Metformin treatment suppresses melanoma cell growth and motility through modulation of microRNA expression. Cancers. (2019) 11:209. 10.3390/cancers11020209 30754729 PMC6406940

[184] Wang Y Dai W Chu X Yang B Zhao M Sun Y. Metformin inhibits lung cancer cells proliferation through repressing microRNA-222. Biotechnol Lett. (2013) 35:2013–9. 10.1007/s10529-013-1309-0 23974492

[185] Blandino G Valerio M Cioce M Mori F Casadei L Pulito C. Metformin elicits anticancer effects through the sequential modulation of DICER and c-MYC. Nat Commun. (2012) 3:865. 10.1038/ncomms1859 22643892

[186] Avci CB Harman E Dodurga Y Susluer SY Gunduz C. Therapeutic potential of an anti-diabetic drug, metformin: alteration of miRNA expression in prostate cancer cells. Asian Pac J Cancer Prev. (2013) 14:765–8. 10.7314/APJCP.2013.14.2.765 23621234

[187] Tsao C-J. microRNA-21-mediated regulation of Sprouty2 protein expression enhances the cytotoxic effect of 5-fluorouracil and metformin in colon cancer cells. Int J Mol Med. (2012) 29:920–6. 10.3892/ijmm.2012.910 22322462

[188] Pulito C Mori F Sacconi A Goeman F Ferraiuolo M Pasanisi P. Metformin-induced ablation of microRNA 21-5p releases Sestrin-1 and CAB39L antitumoral activities. Cell Discov. (2017) 3:17022. 10.1038/celldisc.2017.22 28698800 PMC5501975

[189] Devlin C Greco S Martelli F Ivan M. miR-210: more than a silent player in hypoxia. IUBMB Life. (2011) 63:94–100. 10.1002/iub.427 21360638 PMC4497508

[190] Harting T Stubbendorff M Willenbrock S Wagner S Schadzek P Ngezahayo A. The effect of dichloroacetate in canine prostate adenocarcinomas and transitional cell carcinomas *in vitro*. Int J Oncol. (2016) 49:2341–50. 10.3892/ijo.2016.3720 27748833

[191] Takwi AAL Li Y Becker Buscaglia LE Zhang J Choudhury S Park AK. A statin-regulated microRNA represses human c-Myc expression and function. EMBO Mol Med. (2012) 4:896–909. 10.1002/emmm.201101045 22887866 PMC3491823

[192] Kang M Lee K-H Lee HS Jeong CW Ku JH Kim HH. Concurrent treatment with simvastatin and NF-κB inhibitor in human castration-resistant prostate cancer cells exerts synergistic anti-cancer effects via control of the NF-κB/LIN28/let-7 miRNA signaling pathway. PLoS ONE. (2017) 12:e0184644. 10.1371/journal.pone.0184644 28910332 PMC5599006

[193] Karlic H Thaler R Gerner C Grunt T Proestling K Haider F. Inhibition of the mevalonate pathway affects epigenetic regulation in cancer cells. Cancer Genet. (2015) 208:241–52. 10.1016/j.cancergen.2015.03.008 25978957 PMC4503872

[194] Zheng X Liu K Wang X Zhang R Li X MicroRNA-192 acts as a tumor suppressor in colon cancer and simvastatin activates miR-192 to inhibit cancer cell growth. Mol Med Rep. (2019) 19:1753–60. 10.3892/mmr.2019.9808 30628692

[195] Peng F Wang J-H Fan W-J Meng Y-T Li M-M Li T-T Glycolysis gatekeeper PDK1 reprograms breast cancer stem cells under hypoxia. Oncogene. (2018) 37:1062–74. 10.1038/onc.2017.368 29106390 PMC5851116

[196] Bai F Yu Z Gao X Gong J Fan L Liu F. Simvastatin induces breast cancer cell death through oxidative stress up-regulating miR-140-5p. Aging. (2019) 11:3198–219. 10.18632/aging.101974 31138773 PMC6555469

[197] Bhardwaj A Singh H Trinidad CM Albarracin CT Hunt KK Bedrosian I. The isomiR-140-3p-regulated mevalonic acid pathway as a potential target for prevention of triple negative breast cancer. Breast Cancer Res. (2018) 20:150. 10.1186/s13058-018-1074-z 30537987 PMC6290546

[198] Karataş ÖF Ittmann M MiR-33a and statins collaboratively reduce the proliferative capacity of prostate cancer cells. Eur Res J. (2018) 4:266–74. 10.18621/eurj.380619

[199] Li P Yin Y-L Guo T Sun X-Y Ma H Zhu M-L. Inhibition of aberrant microRNA-133a expression in endothelial cells by statin prevents endothelial dysfunction by targeting GTP cyclohydrolase 1 *in vivo*. Circulation. (2016) 134:1752–65. 10.1161/CIRCULATIONAHA.116.017949 27765794 PMC5120771

[200] Lam HC Liu H-J Baglini CV Filippakis H Alesi N Nijmeh J. Rapamycin-induced miR-21 promotes mitochondrial homeostasis and adaptation in mTORC1 activated cells. Oncotarget. (2017) 8:64714–27. 10.18632/oncotarget.19947 29029388 PMC5630288

[201] Totary-Jain H Sanoudou D Ben-Dov IZ Dautriche CN Guarnieri P Marx SO. Reprogramming of the MicroRNA transcriptome mediates resistance to rapamycin. J Biol Chem. (2013) 288:6034–44. 10.1074/jbc.M112.416446 23300087 PMC3585042

[202] Gan H Lin L Hu N Yang Y Gao Y Pei Y. Aspirin ameliorates lung cancer by targeting the miR-98/WNT1 axis. Thorac Cancer. (2019) 10:744–50. 10.1111/1759-7714.12992 30756509 PMC6449227

[203] Li X Gao L Cui Q Gary BD Dyess DL Taylor W. Sulindac inhibits tumor cell invasion by suppressing NF-κB-mediated transcription of microRNAs. Oncogene. (2012) 31:4979–86. 10.1038/onc.2011.655 22286762 PMC3372649

[204] Kang C. Genome-wide identification of TCF7L2/TCF4 target miRNAs reveals a role for miR-21 in Wnt-driven epithelial cancer. Int J Oncol. (2011) 40:519–26. 10.3892/ijo.2011.1215 21956205

[205] Saito Y Suzuki H Imaeda H Matsuzaki J Hirata K Tsugawa H. The tumor suppressor *microRNA-29c* is downregulated and restored by celecoxib in human gastric cancer cells. Int J Cancer. (2013) 132:1751–60. 10.1002/ijc.27862 23001726

[206] Wang J Zhang X Shi J Cao P Wan M Zhang Q. Fatty acid synthase is a primary target of MiR-15a and MiR-16-1 in breast cancer. Oncotarget. (2016) 7:78566–76. 10.18632/oncotarget.12479 27713175 PMC5346660

[207] Mishra PJ Humeniuk R Mishra PJ Longo-Sorbello GSA Banerjee D Bertino JR. A miR-24 microRNA binding-site polymorphism in dihydrofolate reductase gene leads to methotrexate resistance. Proc Natl Acad Sci USA. (2007) 104:13513–8. 10.1073/pnas.0706217104 17686970 PMC1948927

[208] Song B Wang Y Xi Y Kudo K Bruheim S Botchkina GI. Mechanism of chemoresistance mediated by miR-140 in human osteosarcoma and colon cancer cells. Oncogene. (2009) 28:4065–74. 10.1038/onc.2009.274 19734943 PMC2783211

[209] Song B Wang Y Titmus MA Botchkina G Formentini A Kornmann M. Molecular mechanism of chemoresistance by miR-215 in osteosarcoma and colon cancer cells. Mol Cancer. (2010) 9:96. 10.1186/1476-4598-9-96 20433742 PMC2881118

[210] Valeri N Gasparini P Braconi C Paone A Lovat F Fabbri M. MicroRNA-21 induces resistance to 5-fluorouracil by down-regulating human DNA MutS homolog 2. (hMSH2). Proc Natl Acad Sci USA. (2010) 107:21098–103. 10.1073/pnas.1015541107 21078976 PMC3000294

[211] Akao Y Noguchi S Iio A Kojima K Takagi T Naoe T. Dysregulation of microRNA-34a expression causes drug-resistance to 5-FU in human colon cancer DLD-1 cells. Cancer Lett. (2011) 300:197–204. 10.1016/j.canlet.2010.10.006 21067862

[212] He J Xie G Tong J Peng Y Huang H Li J. Overexpression of microRNA-122 re-sensitizes 5-FU-resistant colon cancer cells to 5-FU through the inhibition of PKM2 *in vitro* and *in vivo*. Cell Biochem Biophys. (2014) 70:1343–50. 10.1007/s12013-014-0062-x 24898807

[213] Shah MY Pan X Fix LN Farwell MA Zhang B. 5-fluorouracil drug alters the microrna expression profiles in MCF-7 breast cancer cells. J Cell Physiol. (2011) 226:1868–78. 10.1002/jcp.22517 21506117

[214] Park J-K Lee EJ Esau C Schmittgen TD Antisense Inhibition of microRNA-21 or−221 arrests cell cycle, induces apoptosis, and sensitizes the effects of gemcitabine in pancreatic adenocarcinoma: *Pancreas*. (2009) 38:e190–9. 10.1097/MPA.0b013e3181ba82e1 19730150

[215] Almeida MI Reis RM Calin GA. Decoy activity through microRNAs: the therapeutic implications. Expert Opin Biol Ther. (2012) 12:1153–9. 10.1517/14712598.2012.693470 22650365 PMC3799811

[216] Kim EJ Tu M-J Duan Z Gonzalez FJ Bold RJ Yu A MicroRNA-1291 effects on pancreatic cancer (PC) cells sensitivity to arginine deprivation and chemotherapy through modulation of ASS1 and GLUT1. J Clin Oncol. (2018) 36:334 10.1200/JCO.2018.36.4_suppl.334

[217] El-Mir M-Y Nogueira V Fontaine E Avéret N Rigoulet M Leverve X. Dimethylbiguanide inhibits cell respiration via an indirect effect targeted on the respiratory chain complex I. J Biol Chem. (2000) 275:223–8. 10.1074/jbc.275.1.223 10617608

[218] Zhou G Myers R Li Y Chen Y Shen X Fenyk-Melody J. Role of AMP-activated protein kinase in mechanism of metformin action. J Clin Invest. (2001) 108:1167–74. 10.1172/JCI13505 11602624 PMC209533

[219] Bridges HR Jones AJY Pollak MN Hirst J. Effects of metformin and other biguanides on oxidative phosphorylation in mitochondria. Biochem J. (2014) 462:475–87. 10.1042/BJ20140620 25017630 PMC4148174

[220] Wheaton WW Weinberg SE Hamanaka RB Soberanes S Sullivan LB Anso E. Metformin inhibits mitochondrial complex I of cancer cells to reduce tumorigenesis. Elife. (2014) 3:e02242. 10.7554/eLife.02242 24843020 PMC4017650

[221] Evans JMM Donnelly LA Emslie-Smith AM Alessi DR Morris AD. Metformin and reduced risk of cancer in diabetic patients. BMJ. (2005) 330:1304–5. 10.1136/bmj.38415.708634.F7 15849206 PMC558205

[222] Lee JH Kim TI Jeon SM Hong SP Cheon JH Kim WH. The effects of metformin on the survival of colorectal cancer patients with diabetes mellitus. Int J Cancer. (2012) 131:752–9. 10.1002/ijc.26421 21913184

[223] Noto H Goto A Tsujimoto T Noda M. Cancer risk in diabetic patients treated with metformin: a systematic review and meta-analysis. PLoS ONE. (2012) 7:e33411. 10.1371/journal.pone.0033411 22448244 PMC3308971

[224] Gandini S Puntoni M Heckman-Stoddard BM Dunn BK Ford L DeCensi A. Metformin and cancer risk and mortality: a systematic review and meta-analysis taking into account biases and confounders. Cancer Prev Res. (2014) 7:867–85. 10.1158/1940-6207.CAPR-13-0424 24985407 PMC4154969

[225] Jiralerspong S Palla SL Giordano SH Meric-Bernstam F Liedtke C Barnett CM. Metformin and pathologic complete responses to neoadjuvant chemotherapy in diabetic patients with breast cancer. J Clin Oncol. (2009) 27:3297–302. 10.1200/JCO.2009.19.6410 19487376 PMC2736070

[226] Gui DY Sullivan LB Luengo A Hosios AM Bush LN Gitego N. Environment dictates dependence on mitochondrial complex I for NAD+ and aspartate production and determines cancer cell sensitivity to metformin. Cell Metab. (2016) 24:716–27. 10.1016/j.cmet.2016.09.006 27746050 PMC5102768

[227] Shaw RJ Kosmatka M Bardeesy N Hurley RL Witters LA DePinho RA. The tumor suppressor LKB1 kinase directly activates AMP-activated kinase and regulates apoptosis in response to energy stress. Proc Natl Acad Sci USA. (2004) 101:3329–35. 10.1073/pnas.0308061100 14985505 PMC373461

[228] Kim HJ Lee S Chun KH Jeon JY Han SJ Kim DJ. Metformin reduces the risk of cancer in patients with type 2 diabetes: an analysis based on the Korean national diabetes program cohort. Medicine. (2018) 97:e0036. 10.1097/MD.0000000000010036 29465545 PMC5841986

[229] Lee M-Y Lin K-D Hsiao P-J Shin S-J. The association of diabetes mellitus with liver, colon, lung, and prostate cancer is independent of hypertension, hyperlipidemia, and gout in Taiwanese patients. Metabolism. (2012) 61:242–9. 10.1016/j.metabol.2011.06.020 21820134

[230] He K Hu H Ye S Wang H Cui R Yi L. The effect of metformin therapy on incidence and prognosis in prostate cancer: a systematic review and meta-analysis. Sci Rep. (2019) 9:2218. 10.1038/s41598-018-38285-w 30778081 PMC6379374

[231] Libby G Donnelly LA Donnan PT Alessi DR Morris AD Evans JMM. New users of metformin are at low risk of incident cancer: a cohort study among people with type 2 diabetes. Diabetes Care. (2009) 32:1620–5. 10.2337/dc08-2175 19564453 PMC2732153

[232] Saunier E Benelli C Bortoli S. The pyruvate dehydrogenase complex in cancer: an old metabolic gatekeeper regulated by new pathways and pharmacological agents: pyruvate dehydrogenase complex in cancer. Int J Cancer. (2016) 138:809–17. 10.1002/ijc.29564 25868605

[233] Kim K Kim JK Han SH Lim J-S Kim KI Cho DH. Adiponectin is a negative regulator of NK cell cytotoxicity. J Immunol. (2006) 176:5958–64. 10.4049/jimmunol.176.10.5958 16670304

[234] Michelakis ED Webster L Mackey JR. Dichloroacetate (DCA) as a potential metabolic-targeting therapy for cancer. Br J Cancer. (2008) 99:989–94. 10.1038/sj.bjc.6604554 18766181 PMC2567082

[235] Wen S Zhu D Huang P. Targeting cancer cell mitochondria as a therapeutic approach. Future Med Chem. (2013) 5:53–67. 10.4155/fmc.12.190 23256813 PMC3587793

[236] Bingham PM Stuart SD Zachar Z. Lipoic acid and lipoic acid analogs in cancer metabolism and chemotherapy. Expert Rev Clin Pharmacol. (2014) 7:837–46. 10.1586/17512433.2014.966816 25284345

[237] Liang Y Hou L Li L Li L Zhu L Wang Y. Dichloroacetate restores colorectal cancer chemosensitivity through the p53/miR-149-3p/PDK2-mediated glucose metabolic pathway. Oncogene. (2019) 10.1038/s41388-019-1035-8 31597953 PMC6949190

[238] Bjarnadottir O Romero Q Bendahl P-O Jirström K Rydén L Loman N. Targeting HMG-CoA reductase with statins in a window-of-opportunity breast cancer trial. Breast Cancer Res Treat. (2013) 138:499–508. 10.1007/s10549-013-2473-6 23471651

[239] Lin JJ Ezer N Sigel K Mhango G Wisnivesky JP. The effect of statins on survival in patients with stage IV lung cancer. Lung Cancer. (2016) 99:137–42. 10.1016/j.lungcan.2016.07.006 27565929 PMC5003323

[240] Hung M-S Chen I-C Lee C-P Huang R-J Chen P-C Tsai Y-H. Statin improves survival in patients with EGFR-TKI lung cancer: a nationwide population-based study. PLoS ONE. (2017) 12:e0171137. 10.1371/journal.pone.0171137 28158206 PMC5291515

[241] Newman A Clutterbuck RD Powles RL Catovsky D Millar JL. A comparison of the effect of the 3-hydroxy-3-methylglutaryl coenzyme A (HMG-CoA) reductase inhibitors simvastatin, lovastatin and pravastatin on leukaemic and normal bone marrow progenitors. Leuk Lymphoma. (1997) 24:533–7. 10.3109/10428199709055590 9086443

[242] Dimitroulakos J Thai S Wasfy GH Hedley DW Minden MD Penn LZ. Lovastatin induces a pronounced differentiation response in acute myeloid leukemias. Leuk Lymphoma. (2000) 40:167–78. 10.3109/10428190009054894 11426618

[243] Denoyelle C. Cerivastatin, an inhibitor of HMG-CoA reductase, inhibits the signaling pathways involved in the invasiveness and metastatic properties of highly invasive breast cancer cell lines: an *in vitro* study. Carcinogenesis. (2001) 22:1139–48. 10.1093/carcin/22.8.1139 11470741

[244] Mannello F Tonti GA. Statins and breast cancer: may matrix metalloproteinase be the missing link. Cancer Invest. (2009) 27:466–70. 10.1080/07357900802491444 19219650

[245] Chiarini F Evangelisti C McCubrey JA Martelli AM. Current treatment strategies for inhibiting mTOR in cancer. Trends Pharmacol Sci. (2015) 36:124–35. 10.1016/j.tips.2014.11.004 25497227

[246] Tolcher AW Bendell JC Papadopoulos KP Burris HA Patnaik A Jones SF. A phase IB trial of the oral MEK inhibitor trametinib (GSK1120212) in combination with everolimus in patients with advanced solid tumors. Ann Oncol. (2015) 26:58–64. 10.1093/annonc/mdu482 25344362

[247] Laplante M Sabatini DM. mTOR signaling in growth control and disease. Cell. (2012) 149:274–93. 10.1016/j.cell.2012.03.017 22500797 PMC3331679

[248] Zaytseva YY Rychahou PG Gao T Lee EY Weiss HL Heuer TS Abstract 1010: evaluation of small-molecule FASN inhibitors in preclinical models of colorectal cancer. Cancer Res. (2016) 76:1010 10.1158/1538-7445.AM2016-1010

[249] Brenner A Falchook G Patel M Infante J Arkenau H-T Dean E Abstract P6-11-09: heavily pre-treated breast cancer patients show promising responses in the first in human study of the first-In-class fatty acid synthase (FASN) inhibitor, TVB-2640 in combination with paclitaxel. Cancer Res. (2017) 77(Suppl. 4). 10.1158/1538-7445.SABCS16-P6-11-09

[250] Zaytseva YY Rychahou PG Le A-T Scott TL Flight RM Kim JT. Preclinical evaluation of novel fatty acid synthase inhibitors in primary colorectal cancer cells and a patient-derived xenograft model of colorectal cancer. Oncotarget. (2018) 9: 24787–800. 10.18632/oncotarget.25361 29872506 PMC5973868

[251] Hundal RS Petersen KF Mayerson AB Randhawa PS Inzucchi S Shoelson SE. Mechanism by which high-dose aspirin improves glucose metabolism in type 2 diabetes. J Clin Invest. (2002) 109:1321–6. 10.1172/JCI0214955 12021247 PMC150979

[252] Paez Espinosa EV Murad JP Khasawneh FT. Aspirin: pharmacology and clinical applications. Thrombosis. (2012) 2012:1–15. 10.1155/2012/173124 22195279 PMC3236360

[253] Hawley SA Fullerton MD Ross FA Schertzer JD Chevtzoff C Walker KJ. The ancient drug salicylate directly activates AMP-activated protein kinase. Science. (2012) 336:918–22. 10.1126/science.1215327 22517326 PMC3399766

[254] Hammerlindl H Ravindran Menon D Hammerlindl S Emran AA Torrano J Sproesser K. Acetylsalicylic acid governs the effect of sorafenib in *RAS* -mutant cancers. Clin Cancer Res. (2018) 24:1090–102. 10.1158/1078-0432.CCR-16-2118 29196297 PMC5844814

[255] Drew DA Cao Y Chan AT. Aspirin and colorectal cancer: the promise of precision chemoprevention. Nat Rev Cancer. (2016) 16:173–86. 10.1038/nrc.2016.4 26868177 PMC6741347

[256] Rothwell PM Fowkes FGR Belch JFF Ogawa H Warlow CP Meade TW. Effect of daily aspirin on long-term risk of death due to cancer: analysis of individual patient data from randomised trials. Lancet Lond Engl. (2011) 377:31–41. 10.1016/S0140-6736(10)62110-1 21144578

[257] Simon TG Ma Y Ludvigsson JF Chong DQ Giovannucci EL Fuchs CS. Association between aspirin use and risk of hepatocellular carcinoma. JAMA Oncol. (2018) 4:1683–90. 10.1001/jamaoncol.2018.4154 30286235 PMC6440745

[258] Barnard ME Poole EM Curhan GC Eliassen AH Rosner BA Terry KL. Association of analgesic use with risk of ovarian cancer in the nurses' health studies. JAMA Oncol. (2018) 4:1675–82. 10.1001/jamaoncol.2018.4149 30286239 PMC6400245

[259] Pinheiro C Longatto-Filho A Scapulatempo C Ferreira L Martins S Pellerin L. Increased expression of monocarboxylate transporters 1, 2, and 4 in colorectal carcinomas. Virchows Arch. (2008) 452:139–46. 10.1007/s00428-007-0558-5 18188595

[260] Pinheiro C Albergaria A Paredes J Sousa B Dufloth R Vieira D Monocarboxylate transporter 1 is up-regulated in basal-like breast carcinoma: MCT1 in breast cancer. Histopathology. (2010) 56:860–7. 10.1111/j.1365-2559.2010.03560.x 20636790

[261] Bola BM Chadwick AL Michopoulos F Blount KG Telfer BA Williams KJ. Inhibition of monocarboxylate transporter-1 (MCT1) by AZD3965 enhances radiosensitivity by reducing lactate transport. Mol Cancer Ther. (2014) 13:2805–16. 10.1158/1535-7163.MCT-13-1091 25281618 PMC4258406

[262] Halford SER Jones P Wedge S Hirschberg S Katugampola S Veal G A first-in-human first-in-class (FIC) trial of the monocarboxylate transporter 1 (MCT1) inhibitor AZD3965 in patients with advanced solid tumours. J Clin Oncol. (2017) 35:2516 10.1200/JCO.2017.35.15_suppl.2516

[263] Mehrmohamadi M Jeong SH Locasale JW. Molecular features that predict the response to antimetabolite chemotherapies. Cancer Metab. (2017) 5:8. 10.1186/s40170-017-0170-3 29026541 PMC5627437

[264] Showalter SL Showalter TN Witkiewicz A Havens R Kennedy EP Hucl T. Evaluating the drug-target relationship between thymidylate synthase expression and tumor response to 5-fluorouracil: Is it time to move forward? Cancer Biol Ther. (2008) 7:986–94. 10.4161/cbt.7.7.6181 18443433 PMC3081718

[265] Costales MG Haga CL Velagapudi SP Childs-Disney JL Phinney DG Disney MD. Small molecule inhibition of microRNA-210 reprograms an oncogenic hypoxic circuit. J Am Chem Soc. (2017) 139:3446–55. 10.1021/jacs.6b11273 28240549 PMC5810126

[266] Kelly TJ Souza AL Clish CB Puigserver P. A hypoxia-induced positive feedback loop promotes hypoxia-inducible factor 1alpha stability through miR-210 suppression of glycerol-3-phosphate dehydrogenase 1-like. Mol Cell Biol. (2011) 31:2696–706. 10.1128/MCB.01242-10 21555452 PMC3133367

[267] Heidelberger C Chaudhuri NK Danneberg P Mooren D Griesbach L Duschinsky R. Fluorinated pyrimidines, a new class of tumour-inhibitory compounds. Nature. (1957) 179:663–6. 10.1038/179663a0 13418758

[268] Wagner AD Grothe W Haerting J Kleber G Grothey A Fleig WE. Chemotherapy in advanced gastric cancer: a systematic review and meta-analysis based on aggregate data. J Clin Oncol. (2006) 24:2903–9. 10.1200/JCO.2005.05.0245 16782930

[269] Parker WB. Enzymology of purine and pyrimidine antimetabolites used in the treatment of cancer. Chem Rev. (2009) 109:2880–93. 10.1021/cr900028p 19476376 PMC2827868

[270] Hanna J Hossain GS Kocerha J. The potential for microRNA therapeutics and clinical research. Front Genet. (2019) 10:478. 10.3389/fgene.2019.00478 31156715 PMC6532434

[271] Garzon R Marcucci G Croce CM. Targeting microRNAs in cancer: rationale, strategies and challenges. Nat Rev Drug Discov. (2010) 9:775–89. 10.1038/nrd3179 20885409 PMC3904431

[272] van Rooij E Kauppinen S. Development of microRNA therapeutics is coming of age. EMBO Mol Med. (2014) 6:851–64. 10.15252/emmm.201100899 24935956 PMC4119351

[273] Shah MY Ferrajoli A Sood AK Lopez-Berestein G Calin GA. microRNA therapeutics in cancer - an emerging concept. EBioMedicine. (2016) 12:34–42. 10.1016/j.ebiom.2016.09.017 27720213 PMC5078622

[274] Yingchoncharoen P Kalinowski DS Richardson DR. Lipid-based drug delivery systems in cancer therapy: what is available and what is yet to come. Pharmacol Rev. (2016) 68:701–87. 10.1124/pr.115.012070 27363439 PMC4931871

[275] Kota J Chivukula RR O'Donnell KA Wentzel EA Montgomery CL Hwang H-W. Therapeutic microRNA delivery suppresses tumorigenesis in a murine liver cancer model. Cell. (2009) 137:1005–17. 10.1016/j.cell.2009.04.021 19524505 PMC2722880

[276] MacDiarmid JA Mugridge NB Weiss JC Phillips L Burn AL Paulin RP. Bacterially derived 400 nm particles for encapsulation and cancer cell targeting of chemotherapeutics. Cancer Cell. (2007) 11:431–45. 10.1016/j.ccr.2007.03.012 17482133

[277] Beg MS Brenner AJ Sachdev J Borad M Kang Y-K Stoudemire J. Phase I study of MRX34, a liposomal miR-34a mimic, administered twice weekly in patients with advanced solid tumors. Invest New Drugs. (2017) 35:180–8. 10.1007/s10637-016-0407-y 27917453 PMC5893501

[278] Reid G Kao SC Pavlakis N Brahmbhatt H MacDiarmid J Clarke S. Clinical development of TargomiRs, a miRNA mimic-based treatment for patients with recurrent thoracic cancer. Epigenomics. (2016) 8:1079–85. 10.2217/epi-2016-0035 27185582

[279] van Zandwijk N Pavlakis N Kao SC Linton A Boyer MJ Clarke S. Safety and activity of microRNA-loaded minicells in patients with recurrent malignant pleural mesothelioma: a first-in-man, phase 1, open-label, dose-escalation study. Lancet Oncol. (2017) 18:1386–96. 10.1016/S1470-2045(17)30621-6 28870611

[280] Seto AG Beatty X Lynch JM Hermreck M Tetzlaff M Duvic M. Cobomarsen, an oligonucleotide inhibitor of miR-155, co-ordinately regulates multiple survival pathways to reduce cellular proliferation and survival in cutaneous T-cell lymphoma. Br J Haematol. (2018) 183:428–44. 10.1111/bjh.15547 30125933

[281] Querfeld C Pacheco T Foss FM Halwani AS Porcu P Seto AG Preliminary results of a phase 1 trial evaluating MRG-106, a synthetic microRNA antagonist (LNA antimiR) of microRNA-155, in patients with CTCL. Blood. (2016) 128:1829 10.1182/blood.V128.22.1829.182927543436

[282] Ghosh D Nandi S Bhattacharjee S. Combination therapy to checkmate Glioblastoma: clinical challenges and advances. Clin Transl Med. (2018) 7:33. 10.1186/s40169-018-0211-8 30327965 PMC6191404

[283] Haque I Banerjee S Mehta S De A Majumder M Mayo MS. Cysteine-rich 61-connective tissue growth factor-nephroblastoma-overexpressed 5 (CCN5)/Wnt-1-induced signaling protein-2 (WISP-2) regulates microRNA-10b via hypoxia-inducible factor-1α-TWIST signaling networks in human breast cancer cells. J Biol Chem. (2011) 286:43475–85. 10.1074/jbc.M111.284158 22020939 PMC3234824

[284] Gomez IG MacKenna DA Johnson BG Kaimal V Roach AM Ren S. Anti-microRNA-21 oligonucleotides prevent Alport nephropathy progression by stimulating metabolic pathways. J Clin Invest. (2015) 125:141–56. 10.1172/JCI75852 25415439 PMC4382246

[285] Steeg PS Theodorescu D. Metastasis: a therapeutic target for cancer. Nat Clin Pract Oncol. (2008) 5:206–19. 10.1038/ncponc1066 18253104 PMC2709494

[286] Chakraborty C Wen Z-H Agoramoorthy G Lin C-S. Therapeutic microRNA delivery strategies with special emphasis on cancer therapy and tumorigenesis: current trends and future challenges. Curr Drug Metab. (2016) 17:469–77. 10.2174/1389200217666160126142408 26813887

[287] Brannon-Peppas L Blanchette JO. Nanoparticle and targeted systems for cancer therapy. Adv Drug Deliv Rev. (2004) 56:1649–59. 10.1016/j.addr.2004.02.014 15350294

[288] Vander Heiden MG DeBerardinis RJ. Understanding the intersections between metabolism and cancer biology. Cell. (2017) 168:657–69. 10.1016/j.cell.2016.12.039 28187287 PMC5329766

[289] Larsson SC Wolk A. Overweight and obesity and incidence of leukemia: a meta-analysis of cohort studies: overweight and obesity and incidence of leukemia. Int J Cancer. (2008) 122:1418–21. 10.1002/ijc.23176 18027857

[290] Roberts DL Dive C Renehan AG. Biological mechanisms linking obesity and cancer risk: new perspectives. Annu Rev Med. (2010) 61:301–16. 10.1146/annurev.med.080708.082713 19824817

[291] Castillo JJ Reagan JL Ingham RR Furman M Dalia S Merhi B. Obesity but not overweight increases the incidence and mortality of leukemia in adults: a meta-analysis of prospective cohort studies. Leuk Res. (2012) 36:868–75. 10.1016/j.leukres.2011.12.020 22285508

[292] Lehr S Hartwig S Sell H. Adipokines: a treasure trove for the discovery of biomarkers for metabolic disorders. Proteomics Clin Appl. (2012) 6:91–101. 10.1002/prca.201100052 22213627

[293] Arner P Kulyté A. MicroRNA regulatory networks in human adipose tissue and obesity. Nat Rev Endocrinol. (2015) 11:276–88. 10.1038/nrendo.2015.25 25732520

[294] Iacomino G Siani A. Role of microRNAs in obesity and obesity-related diseases. Genes Nutr. (2017) 12:23. 10.1186/s12263-017-0577-z 28974990 PMC5613467

[295] Kasiappan R Rajarajan D. Role of microRNA regulation in obesity-associated breast cancer: nutritional perspectives. Adv Nutr Int Rev J. (2017) 8:868–88. 10.3945/an.117.015800 29141971 PMC5682994

[296] Ayers D Boughanem H Macías-González M. Epigenetic influences in the obesity/colorectal cancer axis: a novel theragnostic avenue. J Oncol. (2019) 2019:7406078. 10.1155/2019/7406078 31007685 PMC6441533

[297] Garrett WS. Cancer and the microbiota. Science. (2015) 348:80–6. 10.1126/science.aaa4972 25838377 PMC5535753

[298] Tsilimigras MCB Fodor A Jobin C. Carcinogenesis and therapeutics: the microbiota perspective. Nat Microbiol. (2017) 2:17008. 10.1038/nmicrobiol.2017.8 28225000 PMC6423540

[299] Elinav E Garrett WS Trinchieri G Wargo J. The cancer microbiome. Nat Rev Cancer. (2019) 19:371–6. 10.1038/s41568-019-0155-3 31186547 PMC6700740

[300] Helmink BA Khan MAW Hermann A Gopalakrishnan V Wargo JA. The microbiome, cancer, and cancer therapy. Nat Med. (2019) 25:377–88. 10.1038/s41591-019-0377-7 30842679

[301] Gill SR Pop M DeBoy RT Eckburg PB Turnbaugh PJ Samuel BS. Metagenomic analysis of the human distal gut microbiome. Science. (2006) 312:1355–9. 10.1126/science.1124234 16741115 PMC3027896

[302] Philipp B. Bacterial degradation of bile salts. Appl Microbiol Biotechnol. (2011) 89:903–15. 10.1007/s00253-010-2998-0 21088832

[303] Schwabe RF Jobin C. The microbiome and cancer. Nat Rev Cancer. (2013) 13:800–12. 10.1038/nrc3610 24132111 PMC3986062

[304] Human Microbiome Project Consortium Structure, function and diversity of the healthy human microbiome. Nature. (2012) 486:207–14. 10.1038/nature11234 22699609 PMC3564958

[305] McDonald D Hyde E Debelius JW Morton JT Gonzalez A Ackermann G. American gut: an open platform for citizen science microbiome research. mSystems. (2018) 3:e00031-18. 10.1128/mSystems.00031-18 29795809 PMC5954204

[306] Francescone R Hou V Grivennikov SI. Microbiome, inflammation, and cancer. Cancer J. (2014) 20:181–9. 10.1097/PPO.0000000000000048 24855005 PMC4112188

[307] Irrazábal T Belcheva A Girardin SE Martin A Philpott DJ. The multifaceted role of the intestinal microbiota in colon cancer. Mol Cell. (2014) 54:309–20. 10.1016/j.molcel.2014.03.039 24766895

[308] Chen J Pitmon E Wang K. Microbiome, inflammation and colorectal cancer. Semin Immunol. (2017) 32:43–53. 10.1016/j.smim.2017.09.006 28982615

[309] Mischke M Plösch T. The gut microbiota and their metabolites: potential implications for the host epigenome. In: Schwiertz A., editor. Microbiota of the Human Body. Cham: Springer International Publishing (2016). p. 33–44. 10.1007/978-3-319-31248-4_3 27161349

[310] Bhat MI Kapila R. Dietary metabolites derived from gut microbiota: critical modulators of epigenetic changes in mammals. Nutr Rev. (2017) 75:374–89. 10.1093/nutrit/nux001 28444216

[311] Ye J Wu W Li Y Li L. Influences of the gut microbiota on DNA methylation and histone modification. Dig Dis Sci. (2017) 62:1155–64. 10.1007/s10620-017-4538-6 28341870

[312] Williams MR Stedtfeld RD Tiedje JM Hashsham SA. MicroRNAs-based inter-domain communication between the host and members of the gut microbiome. Front Microbiol. (2017) 8:1896. 10.3389/fmicb.2017.01896 29021788 PMC5624305

[313] Yuan C Subramanian S. microRNA-mediated tumor–microbiota metabolic interactions in colorectal cancer. DNA Cell Biol. (2019) 38:281–5. 10.1089/dna.2018.4579 30668143 PMC6477581

[314] Warburg O. On the Origin of Cancer Cells. Science. (1956) 123:309–14. 10.1126/science.123.3191.309 13298683

[315] Hu S Liu L Chang EB Wang J-Y Raufman J-P. Butyrate inhibits pro-proliferative miR-92a by diminishing c-Myc-induced miR-17-92a cluster transcription in human colon cancer cells. Mol Cancer. (2015) 14:180. 10.1186/s12943-015-0450-x 26463716 PMC4604099

[316] Dews M Fox JL Hultine S Sundaram P Wang W Liu YY. The Myc-miR-17 92 axis blunts TGF signaling and production of multiple TGF -dependent antiangiogenic factors. Cancer Res. (2010) 70:8233–46. 10.1158/0008-5472.CAN-10-2412 20940405 PMC3007123

[317] Zhang G Zhou H Xiao H Liu Z Tian H Zhou T. MicroRNA-92a functions as an oncogene in colorectal cancer by targeting PTEN. Dig Dis Sci. (2014) 59:98–107. 10.1007/s10620-013-2858-8 24026406

[318] Ke T-W Wei P-L Yeh K-T Chen WT-L Cheng Y-W. MiR-92a promotes cell metastasis of colorectal cancer through PTEN-mediated PI3K/AKT pathway. Ann Surg Oncol. (2015) 22:2649–55. 10.1245/s10434-014-4305-2 25515201

[319] Yuan C Burns MB Subramanian S Blekhman R. Interaction between host microRNAs and the gut microbiota in colorectal cancer. mSystems. (2018) 3:e00205-17. 10.1128/mSystems.00205-17 29795787 PMC5954203

[320] Yang Y Weng W Peng J Hong L Yang L Toiyama Y. *Fusobacterium nucleatum* increases proliferation of colorectal cancer cells and tumor development in mice by activating toll-like receptor 4 signaling to nuclear factor–κB, and up-regulating expression of microRNA-21. Gastroenterology. (2017) 152:851–66.e24. 10.1053/j.gastro.2016.11.018 27876571 PMC5555435

[321] Cougnoux A Dalmasso G Martinez R Buc E Delmas J Gibold L. Bacterial genotoxin colibactin promotes colon tumour growth by inducing a senescence-associated secretory phenotype. Gut. (2014) 63:1932–42. 10.1136/gutjnl-2013-305257 24658599

[322] Dalmasso G Cougnoux A Delmas J Darfeuille-Michaud A Bonnet R. The bacterial genotoxin colibactin promotes colon tumor growth by modifying the tumor microenvironment. Gut Microbes. (2014) 5:675–80. 10.4161/19490976.2014.969989 25483338 PMC4615906

[323] Baek SH. A novel link between SUMO modification and cancer metastasis. Cell Cycle. (2006) 5:1492–5. 10.4161/cc.5.14.3008 16861889

[324] Teng Y Ren Y Sayed M Hu X Lei C Kumar A. Plant-derived exosomal microRNAs shape the gut microbiota. Cell Host Microbe. (2018) 24:637–52.e8. 10.1016/j.chom.2018.10.001 30449315 PMC6746408

[325] Zhang L Hou D Chen X Li D Zhu L Zhang Y. Exogenous plant MIR168a specifically targets mammalian LDLRAP1: evidence of cross-kingdom regulation by microRNA. Cell Res. (2012) 22:107–26. 10.1038/cr.2011.158 21931358 PMC3351925

[326] Izumi H Tsuda M Sato Y Kosaka N Ochiya T Iwamoto H. Bovine milk exosomes contain microRNA and mRNA and are taken up by human macrophages. J Dairy Sci. (2015) 98:2920–33. 10.3168/jds.2014-9076 25726110

